# HIF1α Plays a Crucial Role in the Development of TFE3–Rearranged Renal Cell Carcinoma by Orchestrating a Metabolic Shift Toward Fatty Acid Synthesis

**DOI:** 10.1111/gtc.13195

**Published:** 2025-01-14

**Authors:** Hidekazu Nishizawa, Shintaro Funasaki, Wenjuan Ma, Yoshiaki Kubota, Kazuhide Watanabe, Yuichiro Arima, Shoichiro Kuroda, Takaaki Ito, Mitsuko Furuya, Takanobu Motoshima, Akira Nishiyama, Sally Mehanna, Yorifumi Satou, Hisashi Hasumi, Ryosuke Jikuya, Kazuhide Makiyama, Tomohiko Tamura, Yuichi Oike, Yasuhito Tanaka, Toshio Suda, Laura S. Schmidt, W. Marston Linehan, Masaya Baba, Tomomi Kamba

**Affiliations:** ^1^ Department of Urology, Graduate School of Medical Sciences Kumamoto University Kumamoto Japan; ^2^ Divison of Molecular and Vascular Biology, IRDA Kumamoto University Kumamoto Japan; ^3^ Cambridge Stem Cell Institute, University of Cambridge Cambridge UK; ^4^ Department of Anatomy Institute for Advanced Medical Research and Keio University School of Medicine Tokyo Japan; ^5^ RIKEN Center for Integrative Medical Sciences Yokohama Japan; ^6^ Developmental Cardiology Laboratory, International Research Center for Medical Science (IRCMS) Kumamoto University Kumamoto Japan; ^7^ Department of Medical Technology Kumamoto Health Science University Faculty of Health Sciences Kumamoto Japan; ^8^ Department of Surgical Pathology Hokkaido University Hospital Sapporo Japan; ^9^ Department of Immunology Yokohama City University Graduate School of Medicine Kanagawa Japan; ^10^ Biotechnology Department, Faculty of Nanotechnology for Postgraduate Studies, Cairo University Ad Doqi Egypt; ^11^ Division of Genomics and Transcriptomics, Joint Research Center for Human Retrovirus Infection Kumamoto University Kumamoto Japan; ^12^ Department of Urology Yokohama City University Graduate School of Medicine Kanagawa Japan; ^13^ Advanced Medical Research Center Yokohama City University Kanagawa Japan; ^14^ Department of Molecular Genetics, Graduate School of Medical Sciences Kumamoto University Kumamoto Japan; ^15^ Department of Gastroenterology and Hepatology, Graduate School of Medical Sciences Kumamoto University Kumamoto Japan; ^16^ Laboratory of Stem Cell Regulation, International Research Center for Medical Science (IRCMS) Kumamoto University Kumamoto Japan; ^17^ Urologic Oncology Branch National Cancer Institute, National Institutes of Health Bethesda Maryland USA; ^18^ Basic Science Program, Frederick National Laboratory for Cancer Research National Cancer Institute Frederick Maryland USA

**Keywords:** glycolysis, HIF1α, ketone body production, lipid synthesis, metabolism, SREBP1, TFE3–rearranged renal cell carcinoma

## Abstract

Tumor development often requires cellular adaptation to a unique, high metabolic state; however, the molecular mechanisms that drive such metabolic changes in TFE3–rearranged renal cell carcinoma (TFE3‐RCC) remain poorly understood. TFE3‐RCC, a rare subtype of RCC, is defined by the formation of chimeric proteins involving the transcription factor TFE3. In this study, we analyzed cell lines and genetically engineered mice, demonstrating that the expression of the chimeric protein PRCC‐TFE3 induced a hypoxia‐related signature by transcriptionally upregulating HIF1α and HIF2α. The upregulation of HIF1α by PRCC‐TFE3 led to increased cellular ATP production by enhancing glycolysis, which also supplied substrates for the TCA cycle while maintaining mitochondrial oxidative phosphorylation. We crossed TFE3‐RCC mouse models with *Hif1α* and/or *Hif2α* knockout mice and found that *Hif1α*, rather than *Hif2α*, is essential for tumor development in vivo. RNA‐seq and metabolomic analyses of the kidney tissues from these mice revealed that ketone body production is inversely correlated with tumor development, whereas de novo lipid synthesis is upregulated through the HIF1α/SREBP1‐dependent mechanism in TFE3‐RCC. Our data suggest that the coordinated metabolic shift via the PRCC‐TFE3/HIF1α/SREBP1 axis is a key mechanism by which PRCC‐TFE3 enhances cancer cell metabolism, promoting tumor development in TFE3‐RCC.

## Graphical abstract

This graphical abstract illustrates the key metabolic changes in TFE3–rearranged renal cell carcinoma (TFE3‐RCC). The PRCC‐TFE3 fusion protein upregulates HIF1α, leading to enhanced glycolysis and lipid synthesis through SREBP1 activation. This metabolic shift, characterized by increased glucose uptake, fatty acid synthesis, and reduced ketogenesis, promotes tumor cell growth. The image contrasts the metabolic pathways of TFE3‐RCC with those of normal kidney tissue, highlighting the crucial role of HIF1α in TFE3‐RCC development.

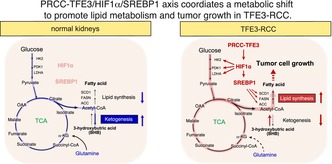

## 1 Introduction

Renal cell carcinoma (RCC) is a heterogeneous cancer, in which 75% are clear cell RCC (ccRCC). The remainder are classified as non‐ccRCC (nccRCC), which includes papillary RCC, chromophobe RCC, TFE3–rearranged RCC (TFE3‐RCC), and other rare types of RCC (Ricketts et al. 2018). The molecular mechanisms underlying RCC are diverse. In sporadic ccRCC, 91% of cases show genetic or epigenetic alterations in the *VHL* gene (Nickerson et al. 2008). This gene encodes pVHL, a component of the E3 ubiquitin ligase. When pVHL is lost, its target proteins HIF1α and HIF2α accumulate excessively, even under normoxic conditions. This leads to abnormal activation of hypoxia signaling pathways (Shirole and Kaelin Jr. 2023). Our in‐depth understanding of the pVHL‐HIF1/2α axis in ccRCC has contributed significantly to the development of molecular‐based therapeutic strategies for advanced ccRCC cases (Choueiri and Kaelin Jr. 2020). In contrast, for nccRCC, a standardized molecular‐based therapy for advanced cases has not yet been established. TFE3‐RCC, in particular, often displays aggressive features and is associated with poor prognosis (Kauffman et al. 2014; Argani 2022). This underscores the urgent need for a deeper understanding of the molecular mechanisms of TFE3‐RCC and the development of targeted therapeutic strategies. TFE3‐RCC is defined in the 2022 WHO classification as a subtype characterized by Xp11.2 rearrangements, leading to *TFE3* gene fusions with various partner genes (Sun et al. 2021; Bakouny et al. 2022; Moch et al. 2022). The chimeric TFE3 protein encoded by these fusions exhibits constitutive transcriptional activity and oncogenic properties (Baba et al. 2019). Both our study and that of another group that expressed the chimeric TFE3 protein in mouse renal epithelial cells demonstrated the formation of TFE3‐RCC and confirmed the oncogenicity of the TFE3 fusion (Baba et al. 2019; Prakasam et al. 2024). The chimeric TFE3 proteins alter a wide range of gene expressions, including those involved in the mTORC1 pathway, E2F pathway, lysosome biogenesis, and redox signaling (Sun et al. 2021; Bakouny et al. 2022; Prakasam et al. 2024). However, the key molecule transcribed by the chimeric TFE3 protein that drives TFE3‐RCC development has yet to be identified. In this study, we have identified HIF1α and HIF2α as direct transcriptional targets of the chimeric TFE3 protein. In ccRCC, HIF1α and HIF2α play oncogenic roles by upregulating hypoxia‐responsive genes, including *HK2*, *PDK1*, *VEGF*, *PDGFB*, *TGFA*, and *CCNC*. Studies using ccRCC cell lines have reported that HIF2α, rather than HIF1α, is important for ccRCC tumorigenicity (Kondo et al. 2003), whereas a recent study using genetically engineered mice has shown that *Hif1α*, not *Hif2α*, is crucial for ccRCC formation (Hoefflin et al. 2020). In this study, we discovered that *Hif1α*, rather than *Hif2α*, plays a crucial role in TFE3‐RCC development, as shown in genetically engineered mice. Additionally, we found that SREBP1, the master regulator of lipid metabolism, is regulated by the PRCC‐TFE3/HIF1α axis. Our findings reveal a novel mechanism whereby a coordinated metabolic shift through the PRCC‐TFE3/HIF1α/SREBP1 axis enhances lipid metabolism, serving as a key step in TFE3‐RCC tumorigenesis.

## 2 Results

### 2.1 PRCC‐TFE3 Expression Upregulates the Hypoxia‐Related Signatures In Vitro and In Vivo

**FIGURE 1:**
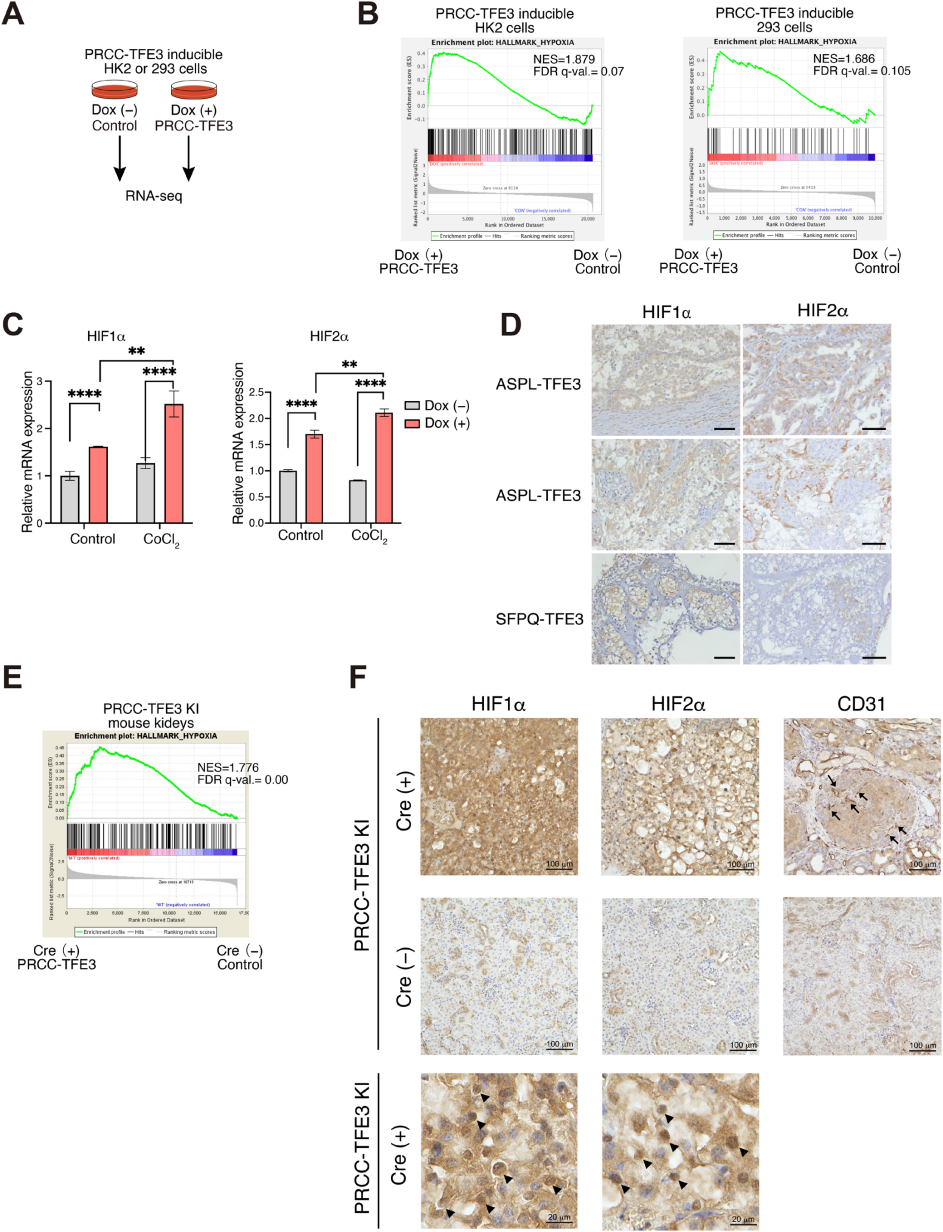
PRCC‐TFE3 expression upregulates hypoxia‐related signatures in vitro and in vivo. (A) RNA‐seq data acquisition using the PRCC‐TFE3–inducible HK2 or HEK293 cell system in a doxycycline (Dox)‐inducible manner. Cells were cultured with or without Dox for 24 h, followed by RNA extraction and gene expression analysis. (B) GSEA plots of differentially expressed genes comparing PRCC‐TFE3–expressing (Dox+) and control (Dox−) cells showed an enrichment of the hypoxia hallmark in both HK2 and HEK293 cells. (C) RT‐qPCR analysis showing HIF1α and HIF2α mRNA levels in PRCC‐TFE3–inducible HK2 cells with or without Dox treatment for 24 h. CoCl₂ treatment (100 μM) was used to simulate hypoxic conditions (*n* = 3). (D) Immunohistochemistry for HIF1α and HIF2α in human TFE3‐RCC samples. Scale bars = 50 μm. (E) GSEA plots of differentially expressed genes comparing PRCC‐TFE3–expressing (Cre+) and control (Cre−) kidneys at 8 months old from the *PRCC‐TFE3* KI mouse model reveal an enrichment for the hypoxia hallmark. (F) Representative staining of HIF1α, HIF2α, and CD31 in kidneys at 8 months from the *PRCC‐TFE3* KI mouse model with or without Cre. Scale bars = 100 μm. Scale bars (magnified images) = 20 μm. Data are means ± SD. *p < 0.05, **p < 0.01, and ****p < 0.0001 (two‐way ANOVA followed by the Sidak post hoc test).

To investigate the downstream mechanisms regulated by the chimeric TFE3 protein, we utilized two kidney cell lines (HK2 and HEK293) that express one of the chimeric TFE3 proteins PRCC‐TFE3 in a doxycycline (Dox)‐dependent manner (Baba et al. 2019; Kurahashi et al. 2019). We performed RNA sequencing (RNA‐seq) on these cells cultured with or without doxycycline (Dox) for 24 h and compared their gene expression profiles (Figure 1A). Gene set enrichment analysis (GSEA) using hallmark gene sets (Liberzon et al. 2015) revealed several significantly enriched signatures upregulated by PRCC‐TFE3 expression (NES > 1.2, *q* value < 0.25), including mTORC1 signaling, protein secretion, and the p53 pathway. These findings confirm that PRCC‐TFE3 regulation in our data aligns with previously identified results in TFE3‐RCC (Figure S1; Sun et al. 2021; Funasaki et al. 2022; Prakasam et al. 2024). Interestingly, we observed that the hypoxia hallmark was enriched with the highest NES in both Dox (+) HK2 and Dox (+) HEK293 cells (Figures 1B and Figure S2), suggesting that the hypoxia‐related pathway is regulated by PRCC‐TFE3 expression. These results lead us to hypothesize that PRCC‐TFE3 controls hypoxia‐related pathway. The hypoxia‐related pathway is known to be regulated by the transcription factors HIF1α and HIF2α, which contribute to tumor development in various cancers including ccRCC (Kaelin Jr. and Ratcliffe 2008; Semenza 2012a). HIF1α and HIF2α are degraded under normoxic conditions through proline hydroxylation, which is recognized and ubiquitinated by the von Hippel‐Lindau (VHL) E3 ligase (Kamura et al. 1999; Stebbins, Kaelin Jr., and Pavletich 1999; Semenza 2014). Under hypoxic conditions, the activity of prolyl hydroxylase domain‐containing proteins (PHDs) is inhibited, leading to the accumulation of HIF1α and HIF2α protein (Schofield and Ratcliffe 2004; Fong and Takeda 2008; Jaakkola et al. 2001). In ccRCC, the high frequency of *VHL* mutations and 3p deletions leads to the accumulation of HIF proteins, which contribute to tumor development (Sato et al. 2013; Gossage, Eisen, and Maher 2015; Mitchell et al. 2018). We found that induction of PRCC‐TFE3 expression upregulates HIF1α and HIF2α mRNA levels in HK2 cells (Figure 1C). These expression levels further increased under hypoxia‐mimetic conditions (CoCl_2_), suggesting that the hypoxia‐related signature caused by PRCC‐TFE3 expression is regulated through the induction of HIF1α and HIF2α (Figure 1C). However, because post‐translational modifications and proteolysis are the primary regulators of HIF1/2α transcriptional activity, additional validation is needed to confirm whether the observed mRNA upregulation translates into increased HIF activity. Indeed, certain HIF1α‐ and HIF2α‐specific target genes are upregulated following PRCC‐TFE3 induction in both HK2 and HEK293 cells (Figure S3). As expected, we also observed HIF1α and HIF2α protein expression in tumors from multiple TFE3‐RCC clinical cases (Figure 1D). To further characterize PRCC‐TFE3 regulation in vivo, we analyzed gene expression profiles from our PRCC‐TFE3–expressing TFE3‐RCC mouse model (Baba et al. 2019; Funasaki et al. 2022) and confirmed that the hypoxia hallmark is enriched in the PRCC‐TFE3–expressing kidneys (Figures 1E and Figure S4). In addition, HIF1α and HIF2α expression was observed in PRCC‐TFE3–expressing tumor regions, which also contained many CD31‐positive cells, indicating hypervascularity (Figure 1F). Taken together, these results suggested that PRCC‐TFE3 modulates HIF1α, HIF2α, and hypoxia‐related signatures both in vitro and in vivo.

### 2.2 PRCC‐TFE3 Directly Binds to the Promoters of *HIF1α* and *HIF2α* , Regulating Their Expression

**FIGURE 2:**
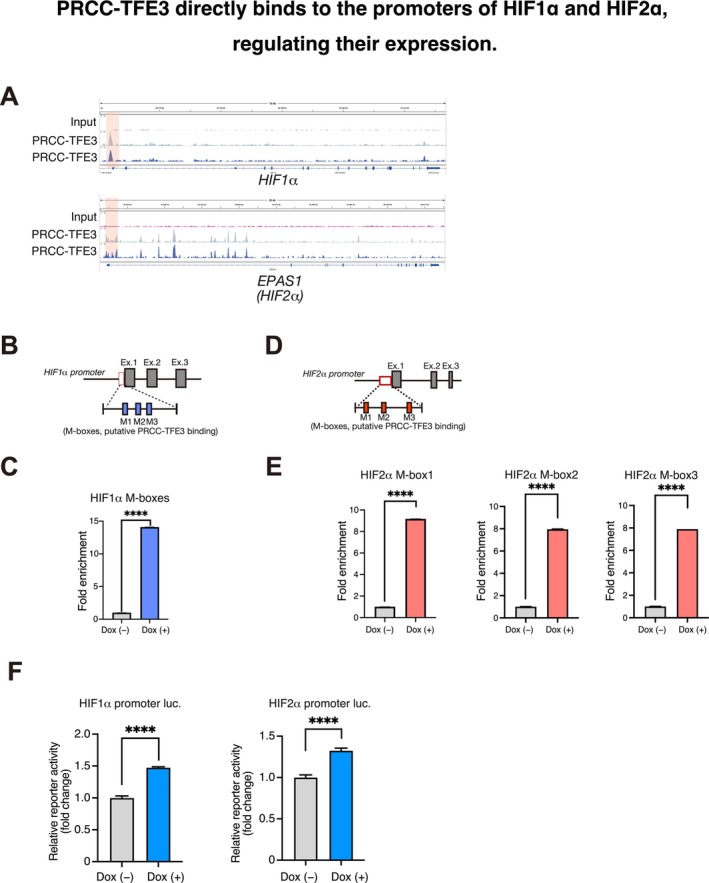
PRCC‐TFE3 directly binds to the promoters of *HIF1α* and *HIF2α*, regulating their expression. (A) ChIP‐seq with HA antibody in HA‐PRCC‐TFE3–induced HK2 cells showing a peak upstream of the transcriptional start sites of *HIF1α* and *HIF2α*. (B) Schematic diagram showing the location of the *HIF1α* promoter with putative PRCC‐TFE3‐binding M‐boxes. (C) ChIP‐qPCR analysis was conducted using primers designed to amplify the putative PRCC‐TFE3–binding M‐boxes in the *HIF1α* promoter region, as shown in (B). HA‐PRCC‐TFE3–inducible HK2 cells were treated with or without Dox for 24 h. Following ChIP of HA‐PRCC‐TFE3, qPCR analysis was performed on the *HIF1α* M‐box region (*n* = 3). (D) Schematic diagram showing the location of the *HIF2α* promoter with putative PRCC‐TFE3–binding M‐boxes, as in (B). (E) ChIP‐qPCR analysis was performed using primers designed to amplify the putative PRCC‐TFE3–binding M‐boxes in the *HIF2α* promoter regions, as shown in (D). Following chromatin immunoprecipitation of HA‐PRCC‐TFE3, qPCR analysis was conducted on the *HIF2α* M‐box region (*n* = 3), as shown in (C). (F) Luciferase reporter assay using HA‐PRCC‐TFE3–inducible HEK293 cells expressing promoter fragments from the 5′ regions of the human *HIF1α* (left) or *HIF2α* gene (right) after 24 h of Dox treatment. Luciferase activity was quantified using a Dual‐Luciferase Reporter Assay System (*n* = 3). Data are means ± SD. (****p < 0.0001, Welch's *t*‐test.)

Next, we investigated the mechanisms by which PRCC‐TFE3 regulates HIF1α and HIF2α expression. Chromatin immunoprecipitation sequencing (ChIP‐seq) using hemagglutinin (HA) tag antibodies revealed binding peaks of HA‐PRCC‐TFE3 in the promoter regions of *HIF1α* and *HIF2α* (Figure 2A), indicating that PRCC‐TFE3 directly controls their transcription. The MiTF/TFE family of transcription factors has been shown to interact with M‐box DNA sequences (TCAYRTGA) within its transcriptional target genes (Napolitano and Ballabio 2016; Slade and Pulinilkunnil 2017). We identified three M‐boxes in the promoters of both *HIF1α* and *HIF2α*, spanning 91 bases and 1124 bases, respectively (Figure 2B,D). ChIP‐qPCR analysis confirmed that PRCC‐TFE3 binds to these promoter regions of *HIF1α* and *HIF2α* following Dox treatment in PRCC‐TFE3–inducible HK2 cells (Figure 2C,E).

To further validate that PRCC‐TFE3 binding to the promoters of *HIF1α* and *HIF2α* drives transcriptional activity, we designed a reporter vector containing the putative PRCC‐TFE3 binding regions upstream of the luciferase gene. Dox treatment enhanced the luciferase activities of both *HIF1α* and *HIF2α* promoter reporters in PRCC‐TFE3–inducible HEK293 cells (Figure 2F). Thus, these results suggest that PRCC‐TFE3 directly binds to the *HIF1α* and *HIF2α* promoters, upregulating their mRNA expression in TFE3‐RCC. Given that aberrant HIFα activation contributes to tumor development in ccRCC, our data suggest a similar role for HIFα expression in TFE3‐RCC, though it involves direct transcriptional regulation rather than effects on protein stability.

### 2.3 HIF1α Regulates Both Glycolysis and Oxidative Phosphorylation in PRCC‐TFE3–Expressing Cells

**FIGURE 3:**
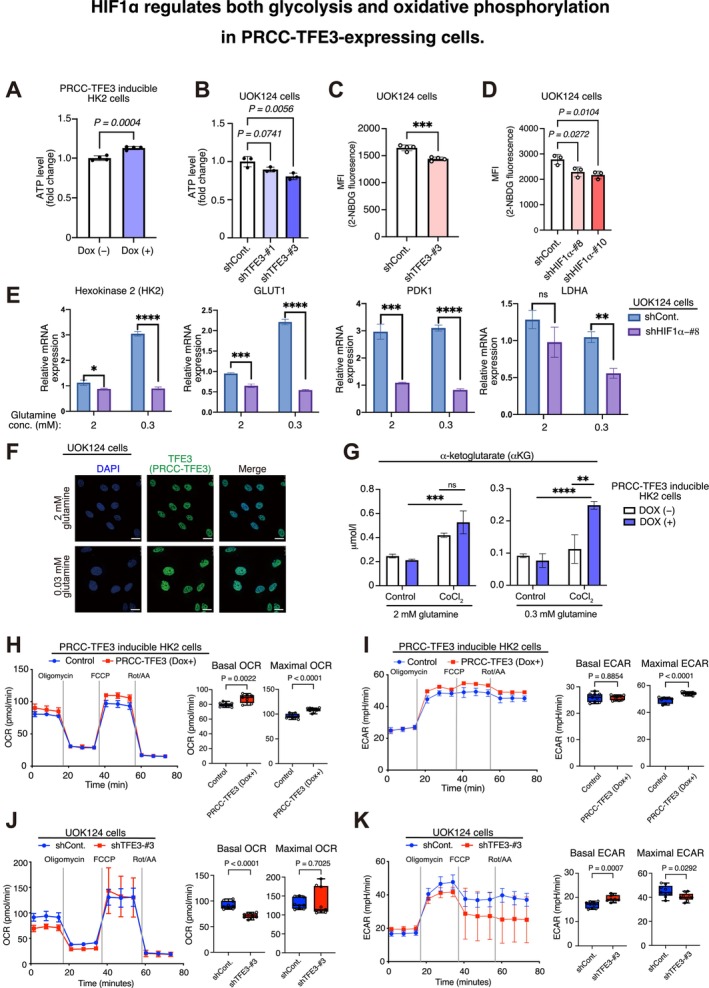
HIF1α regulates both glycolysis and oxidative phosphorylation in PRCC‐TFE3–expressing cells. (A, B) Fold changes in ATP levels were measured in PRCC‐TFE3–inducible HK2 cells with or without Dox treatment for 24 h (A), and in UOK124 cells stably expressing either a control shRNA vector (shCont.) or shRNA targeting PRCC‐TFE3 (shTFE3‐#1 or shTFE3‐#3) (B), 24 h after seeding, using the CellTiter‐Glo luminescent cell viability assay. (C, D) Flow cytometry analysis of glucose uptake using 2‐NBDG incorporation in UOK124 cells expressing shControl or shTFE3‐#3, as well as in cells with control shRNA and shRNA targeting HIF1 α (shHIF1α‐#8 and shHIF1α‐#10). (D) MFI, mean fluorescent intensity (*n* = 3). (E) RT‐qPCR analyses of glycolysis‐related genes in UOK124 cells expressing shCont. or shHIF1α, cultured under different glutamine concentrations (2 or 0.3 mM glutamine) for 24 h (*n* = 3). (F) UOK124 cells were cultured for 24 h in 2 or 0.03 mM glutamine, and PRCC‐TFE3 expression was analyzed by immunofluorescence using anti‐TFE3 antibodies. Scale bar = 10 μm. (G) PRCC‐TFE3–inducible HK2 cells were cultured with or without CoCl_2_ (100 μM, hypoxia‐mimetic reagent) under high (2 mM) or low (0.3 mM) glutamine conditions. α‐Ketoglutarate concentrations were analyzed after 24 h of Dox treatment (*n* = 3). (H, I) Oxygen consumption rate (OCR) (H) and extracellular acidification rate (ECAR) (I) in PRCC‐TFE3–inducible HK2 cells cultured with or without Dox for 24 h, measured using the mitochondrial stress test on the Agilent Seahorse XF HS Mini Analyzer (*n* = 3). Quantitative analysis of the basal and maximal steady states of OCR and ECAR are shown on the left. (J, K) OCR (J) and ECAR (K) in UOK124 cells, measured using the mitochondrial stress test on the Agilent Seahorse XF HS Mini Analyzer (*n* = 3). Quantitative analysis of the basal and maximal steady states of OCR and ECAR are shown on the left. Data are means ± SD. *p < 0.05, **p < 0.01, ***p < 0.01, and ****p < 0.0001; ns, not significant (Welch's t‐test, one‐way ANOVA, or the two‐way ANOVA followed by the Dunnett post hoc test.)

The enhanced expression of HIF1α and HIF2α leads us to hypothesize a global metabolic shift in PRCC‐TFE3–expressing cells. In ccRCC, among stabilized HIFs, HIF1α, rather than HIF2α, contributes to early metabolic changes by upregulating glycolysis, which is essential for tumorigenesis (Hoefflin et al. 2020). Therefore, we focused on HIF1α in this study because, although HIF2α may also play a role, previous reports highlight HIF1α as the dominant factor initiating glycolytic reprogramming in RCC. In HK2 cells, PRCC‐TFE3 induction with Dox treatment caused the elevation of ATP levels (Figure 3A). ATP production also depends on PRCC‐TFE3 expression, as PRCC‐TFE3 knockdown significantly reduced ATP levels in the patient‐derived TFE3‐RCC cell line UOK124 (Figure 3B). Using a fluorescent‐labeled deoxyglucose analog (2‐[*N*‐(7‐nitrobenz‐2‐oxa‐1,3‐diazol‐4‐yl) amino]‐2‐deoxy‐d‐glucose [2‐NBDG]), we found that PRCC‐TFE3 knockdown reduced glycolytic activity in UOK124 cells (Figure 3C). HIF1α has been shown to target various downstream genes that regulate glycolysis under hypoxic conditions (Kierans and Taylor 2021). As expected, HIF1α knockdown also impaired glucose metabolism in UOK124 cells (Figures 3D and Figure S5A), suggesting that HIF1α is primarily responsible for the increased glycolytic activity that generates more ATP in PRCC‐TFE3–expressing cells. Nevertheless, we cannot exclude the possibility that HIF2α also contributes to enhancing glucose uptake, even though HIF1α appears to be the dominant factor. RT‐qPCR analyses revealed that UOK124 cells express multiple HIF1α downstream genes involved in glycolysis (*HK2*, *GLUT1*, *PDK1*, and *LDHA*), all of which were suppressed by HIF1α knockdown (Figure 3E). Interestingly, the effect of PRCC‐TFE3 on the expression of HIF1α downstream genes is more pronounced under low glutamine culture conditions (Figure 3E). We found that GPNMB mRNA expression, a direct target of PRCC‐TFE3, increases under low glutamine conditions but not under low glucose conditions (Figure S5), indicating that PRCC‐TFE3 activity is enhanced under reduced glutamine levels. Low glutamine concentration increases PRCC‐TFE3 expression in UOK124 cells (Figure 3F). These results suggest that PRCC‐TFE3 modulates glucose metabolism, with its role becoming more critical under low glutamine conditions.

Glucose and glutamine metabolism support the tricarboxylic acid (TCA) cycle by supplying substrates for mitochondrial oxidation to carbon dioxide in cells (Figure S5). Given that HIF1α transcription increases glycolysis, with further enhancement under low glutamine conditions, we hypothesized that PRCC‐TFE3 expression shifts cells to prioritize TCA cycle substrates from glucose rather than glutamine. Although we did not observe a significant change in α‐KG concentration between PRCC‐TFE3 non‐induced and induced HK2 cells under high glutamine levels (2 mM), we found that α‐KG concentration significantly increased in PRCC‐TFE3–induced HK2 cells at low glutamine levels (0.3 mM) under hypoxia‐mimetic conditions (CoCl_2_; Figure 3G). These results suggest that PRCC‐TFE3 may enhance glucose utilization for energy production, potentially supporting glycolysis and providing substrates for the TCA cycle. Nevertheless, because multiple pathways depend on α‐KG, interpreting these changes as a global metabolic shift requires careful consideration. Finally, we validated this hypothesis by analyzing mitochondrial metabolic flux with the Seahorse XFe analyzer measuring the oxygen consumption rate (OCR) and extracellular acidification rate (ECAR). PRCC‐TFE3 expression increased not only glucose metabolism (ECAR) but also the OCR in HK2 cells (Figure 3H,I). Conversely, inhibition of PRCC‐TFE3 significantly reduced maximal ECAR and basal OCR in UOK124 cells (Figure 3J,K). Thus, these data suggest that the PRCC‐TFE3/HIF1α axis systemically enhances glucose metabolism, accompanied by sustained mitochondrial activity.

### 2.4 *Hif1α* Knockout, but Not *Hif2α* Knockout, Inhibits Tumor Development in an TFE3‐RCC Mouse Model

**FIGURE 4:**
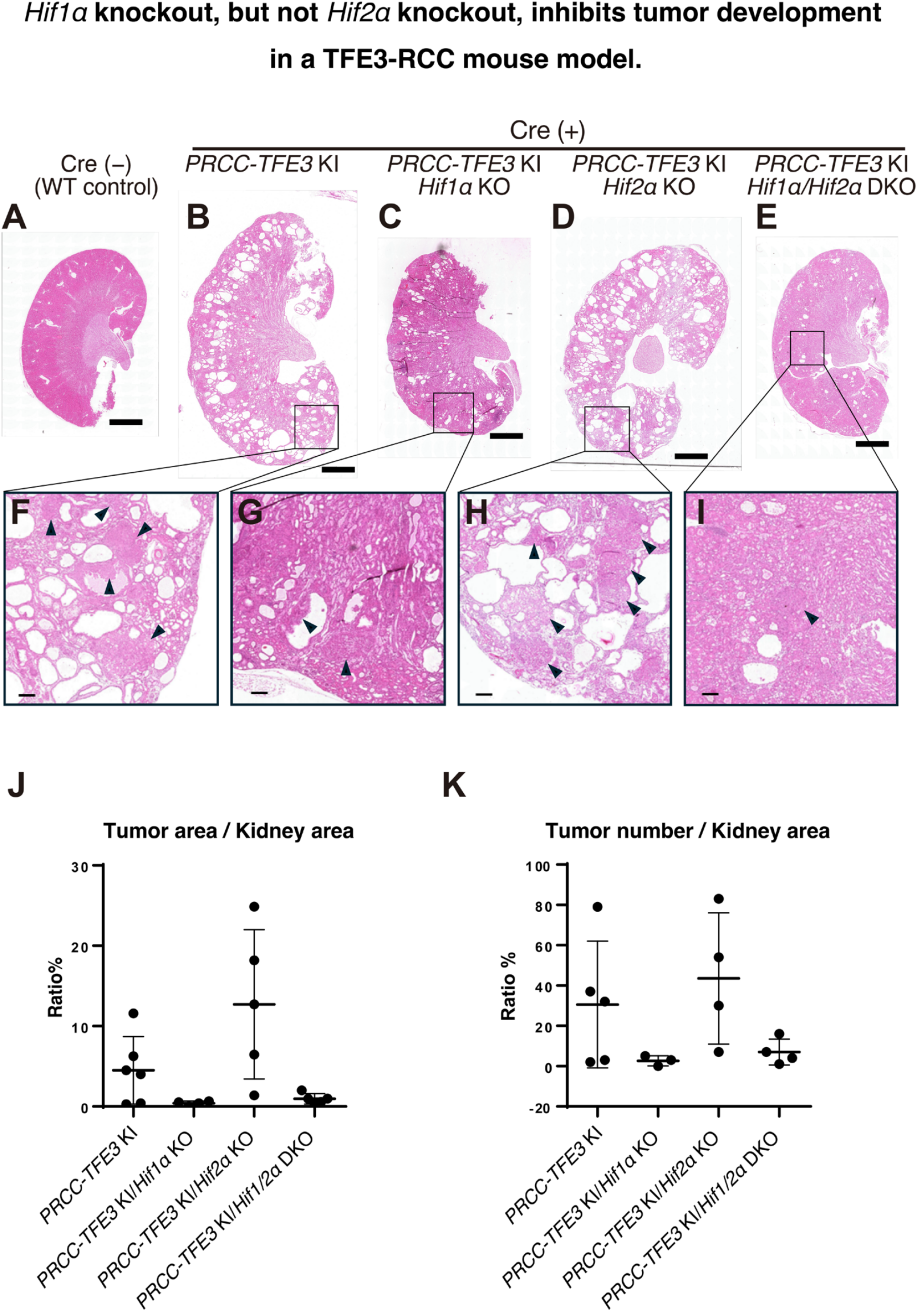
*Hif1α* knockout, but not *Hif2α* knockout, inhibits tumor development in a TFE3‐RCC mouse model. *Rosa26‐lSl‐PRCC‐TFE3* KI mice (*PRCC‐TFE3* KI) were crossed with *Cadherin 16‐Cre* (Cre) transgenic mice and either *Hif1α* ^*f/f*^ (*HIF1α* KO), *Hif2α* ^*f/f*^ (*HIF2α* KO), or *Hif1α* ^*f/f*^ *Hif2α* ^*f/f*^ (*HIF1α/HIF2α* DKO) mice. The mice were sacrificed at 8 months of age, and their kidneys were dissected and subjected to H&E staining. (A) *PRCC‐TFE3* KI without Cre (Cre(−)), (B, F) *PRCC‐TFE3* KI, (C, G) *PRCC‐TFE3* KI/*Hif1*α KO, (D, H) *PRCC‐TFE3* KI/*Hif2α* KO, (E, I) *PRCC‐TFE3* KI/*Hif1α*/*Hif2α* DKO. Scale bars = 2 mm (A–E) and 200 μm (F–I). (J) Tumor areas were measured using a BZ‐X700 microscope (Keyence), and the tumor‐to‐kidney area ratios were then calculated (*n* = 6: *PRCC‐TFE3* KI; *n* = 4: *PRCC‐TFE3* KI/*Hif1α* KO; *n* = 5: *PRCC‐TFE3* KI/*Hif2α* KO; *n* = 5: *PRCC‐TFE3* KI/*Hif1α*/*Hif2α* DKO). (K) Tumor counts were performed using a BZ‐X700 microscope (Keyence) (*n* = 6: *PRCC‐TFE3* KI; *n* = 4: *PRCC‐TFE3* KI/*Hif1α* KO; *n* = 5: *PRCC‐TFE3* KI/*Hif2α* KO; *n* = 5: *PRCC‐TFE3* KI/*Hif1α*/*Hif2α* DKO).

Given that PRCC‐TFE3 upregulates HIF1α and HIF2α to regulate hypoxia‐related signatures in vivo (Figure 1E,F), we next investigated whether Hif1α and/or Hif2α are essential for tumor development. We previously reported on a TFE3‐RCC mouse model in which PRCC‐TFE3 is artificially expressed in kidney epithelial cells by crossing *Rosa26‐lSl‐PRCC‐TFE3* knockin (KI) mice with *Cadherin 16‐Cre* (Cre) mice. (Baba et al. 2019). We crossed these mice with *Hif1α* ^*f/f*^ (*Hif1α* knockout [KO]), *Hif2α* ^*f/f*^ (*Hif2α* KO), and both *Hif1α* ^*f/f*^ *and Hif2α* ^*f/f*^ (*Hif1α*/*Hif2α* double KO [DKO]) mice to analyze tumor development in the kidneys (Figure 4A–I). After 8 months, tumor development was observed in the kidneys of *PRCC‐TFE3* KI mice (Figure 4B,F). However, there was an observed reduction in tumor development in the *PRCC‐TFE3* KI/*Hif1α* KO group (Figure 4C,G) and *PRCC‐TFE3* KI/*Hif1α*/*Hif2α* DKO group (Figure 4E,I). No inhibitory effect was observed in the *PRCC‐TFE3* KI/*Hif2α* KO (Figure 4D,H). Quantification of tumor area and number per kidney further supported that *PRCC‐TFE3* KI/*Hif1α* KO tended to inhibit tumor development (Figure 4J,K). Although *PRCC‐TFE3* KI/*Hif1α* KO showed a reduction in both tumor area and number, cystic tubular dilation was still observed in the tissue, suggesting that *Hif1α* KO does not completely reverse the pathological changes in the kidneys of *PRCC‐TFE3* KI (Figure 4C,G). Overall, our data suggest that only *Hif1α*, and not *Hif2α*, plays a critical role in PRCC‐TFE3–induced tumor development in vivo.

### 2.5 Ketone Metabolism Is Inversely Correlated With Tumor Development in the TFE3‐RCC Mouse Model

**FIGURE 5:**
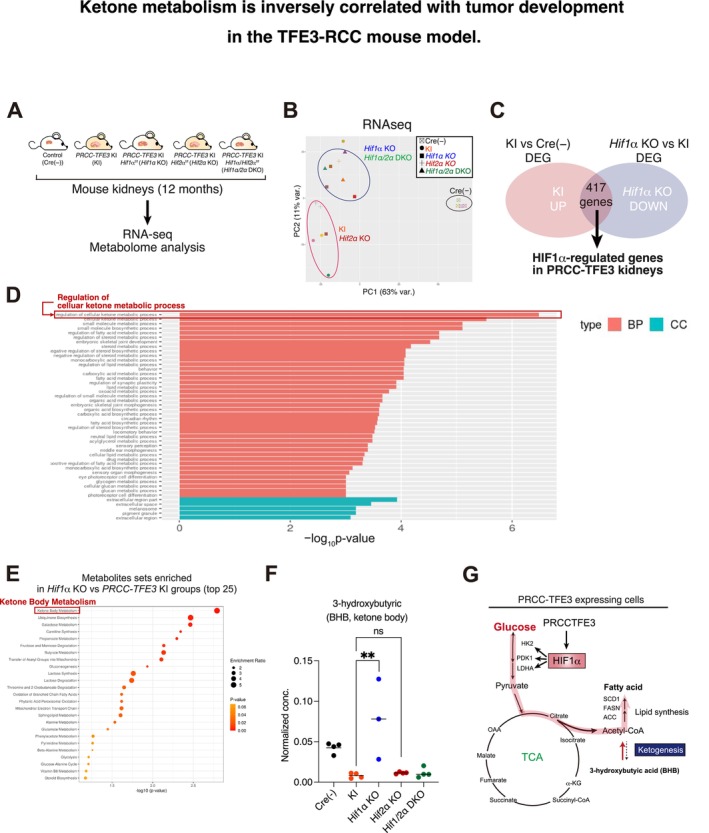
Ketone metabolism is inversely correlated with tumor development in the TFE3‐RCC mouse model. (A) Schematic diagram illustrating the kidney samples from mouse models used for RNA‐seq and metabolomic analyses. (B) PCA analysis of the gene expression data from RNA‐seq, as shown in (A). (C) HIF1α–regulated genes in PRCC‐TFE3–expressing kidneys were identified from genes that were downregulated in *PRCC‐TFE3* KI/*HIF1α* KO compared to *PRCC‐TFE3* KI, and from genes that were upregulated in *PRCC‐TFE3* KI compared to Cre (−) control (*n* = 3–4). (D) Gene ontology analysis of the 417 genes identified in (C), showing enriched terms compared to the mouse whole genome. BP, biological process; CC, cellular component. (E) Enrichment analysis of metabolites in *PRCC‐TFE3* KI/*Hif1α* KO compared to *PRCC‐TFE3* KI groups. (F) Normalized concentration of 3‐hydroxybutyric acid (BHB), a differentially regulated metabolite associated with ketone body metabolism, from the mouse metabolome data in (A) (*n* = 3–4). (G) Schematic diagram illustrating glucose as a potential source of substrates for the TCA cycle and fatty acid synthesis. Data are means ± SD. **p < 0.01; ns, not significant (the one‐way ANOVA followed by the Sidak post hoc test).

To further analyze the downstream mechanisms by which PRCC‐TFE3 regulates tumor development via HIF1α, we performed RNA‐seq analysis on kidney tissues from TFE3‐RCC mice (Figure 5A). Principal component analysis (PCA) of the gene expression data revealed that *PRCC‐TFE3* KI/*Hif2α* KO kidneys exhibited a similar expression profile to those of *PRCC‐TFE3* KI kidneys, whereas *PRCC‐TFE3* KI/*Hif1α* KO and *PRCC‐TFE3* KI/*Hif1α*/*Hif2α* DKO kidneys formed distinct clusters (Figure 5B). This finding aligns with the observation that *Hif1α* is responsible for tumor development in vivo (Figure 4A). To characterize the downstream pathways regulated by HIF1α, we analyzed genes that were upregulated in *PRCC‐TFE3* KI kidneys and downregulated by *Hif1α* KO, identifying a total of 417 such genes (Figure 5C). Gene ontology analysis of this 417‐gene set identified a significantly altered signature, with “regulation of cellular ketone metabolic process” emerging as a top hit (Figure 5D). Furthermore, metabolome analysis of these kidney samples revealed similar global trends in the PCA analysis (Figure S6). The ketone body metabolism signature was also enriched as a top hit in the metabolome comparison between *PRCC‐TFE3* KI/*Hif1α* KO and *PRCC‐TFE3* KI, highlighting the role of HIF1α in the ketone metabolic process in PRCC‐TFE3 kidneys (Figure 5E). Of the 200 metabolites quantified in our analysis, 16 were upregulated and 1 was downregulated in *PRCC‐TFE3* KI/*Hif1α* KO compared to *PRCC‐TFE3* KI (*p* < 0.05, |log_2_ fold change < 0.58; Figure S6). One of the most significantly upregulated metabolites was 3‐hydroxybutyric acid (BHB), a ketone body synthesized from acetyl‐CoA (Mierziak, Burgberger, and Wojtasik 2021; Figure S6). Indeed, BHB concentration decreased in *PRCC‐TFE3* KI and *PRCC‐TFE3 KI*/*Hif2α* KO kidneys, whereas it was either increased or maintained in *PRCC‐TFE3* KI/*Hif1α* KO and *PRCC‐TFE3* KI/*Hif1α*/*Hif2α* DKO kidneys (Figure 5F), suggesting that BHB production is inversely correlated with tumor development.

Collectively, these findings indicate that HIF1α–mediated transcriptional regulation drives metabolic shifts resulting in reduced ketone body levels in TFE3‐RCC. HIF1α is known to shift intracellular fatty acid metabolism from β‐oxidation toward lipid synthesis (Huang et al. 2014), which may contribute to the suppression of ketogenesis in TFE3‐RCC. When ketogenesis is suppressed, cells may redirect acetyl‐CoA to other metabolic pathways, including the TCA cycle and fatty acid synthesis. Conversely, in the absence of HIF1α, the decrease in fatty acid synthesis and the increase in β‐oxidation can free acetyl‐CoA for ketone body production, which may lead to elevated BHB levels. Therefore, we hypothesized that the higher BHB levels might be associated with reduced tumor development and provide a foundation for our analysis of the lipid synthesis pathways (Figure 5G).

### 2.6 HIF1α Regulates SREBP1 Expression, Controlling Lipid Synthesis Pathways and Promoting Cancer Cell Growth

**FIGURE 6:**
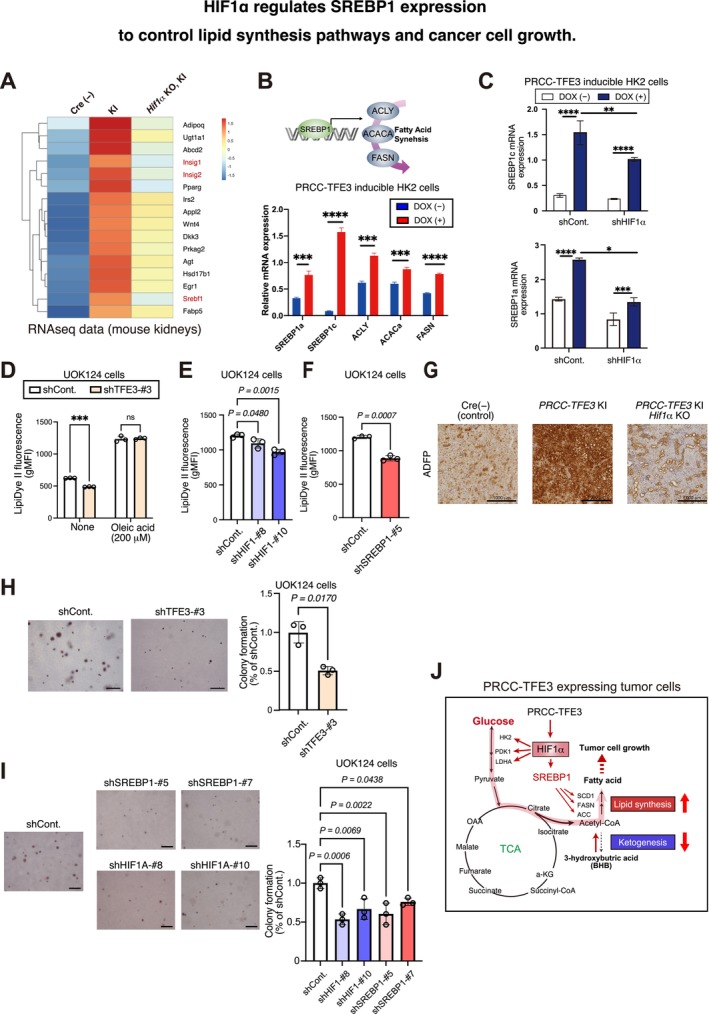
HIF1α regulates SREBP1 expression to control lipid synthesis pathways and cancer cell growth. (A) Heatmap of the top hits from the *PRCC‐TFE3* KI upregulated and *PRCC‐TFE3* KI/*Hif1α* ΚΟ downregulated genes list (417 genes) associated with lipid synthesis. (B) RT‐qPCR analysis of the genes related to lipid synthesis in PRCC‐TFE3–inducible HK2 cells with or without doxycycline treatment for 24 h (*n* = 3). (C) RT‐qPCR analyses of SREBP1a and SREBP1c in PRCC‐TFE3–inducible HK2 cells expressing shCont. or shHIF1α, with or without Dox treatment for 24 h (*n* = 3). (D–F) Flow cytometry analysis of lipid droplets in UOK124 cells using LipidDye II fluorescence: Quantified in cells expressing shCont. or shTFE3 (D), shCont. or HIF1α knockdown (shHIF1α‐#8 and shHIF1α‐#10) (E), and shCont. or SREBP1 knockdown (shSREBP1‐#5) (F). gMFI, geometric mean fluorescence intensity. *n* = 3. (G) Representative staining of adipose differentiation–related protein (ADFP) in the kidneys at 8 months from *PRCC‐TFE3* KI, Cre(−) (WT), *PRCC‐TFE3* KI, and *PRCC‐TFE3* KI/*Hif1*α KO mice. Scale bar = 1000 μm. (H, I) Anchorage‐independent growth analysis of UOK124 cells expressing shTFE3 (H) or shHIF1α and shSREBP1 (I) cultured for 21 days. Scale bar = 500 μm (*n* = 3). (J) Schematic diagram illustrating the PRCC‐TFE3/HIF1α/SREBP1 axis cooperatively regulating the shift of glucose utilization toward glycolysis and fatty acid synthesis. Data are means ± SD. *p < 0.05, **p < 0.01, ***p < 0.01, and ****p < 0.0001; ns, not significant (Welch's t‐test, one‐way ANOVA followed by the Dunnett post hoc test, or the two‐way ANOVA followed by the Sidak post hoc test).

The reduced ketogenesis observed in PRCC‐TFE3–expressing kidneys and its reversal in *PRCC‐TFE3* KI/*Hif1α* KO led us to investigate whether the *PRCC‐TFE3*/*Hif1α* axis is responsible for fatty acid synthesis in TFE3‐RCC. Consistent with this idea, the HIF1 α–regulated gene set (Figure 5C) involved in ketone metabolism includes several genes associated with lipid synthesis (Figure S7). One such gene is *Srebf1*, which encodes Srebp1, a master regulator of lipid synthesis, along with its direct target genes *Insig1* and *Insig2* (Yabe, Brown, and Goldstein 2002; Figure 6A). Indeed, RNA‐seq data showed that the mRNA expressions of Srebp1 and Insig1/2 correlate with *PRCC‐TFE3* KI and are inhibited in *PRCC‐TFE3* KI/*Hif1α* KO (Figure 6A), whereas Srebp1 expression is not suppressed in *PRCC‐TFE3 KI*/*Hif2α* KO (Figure S8). SREBP1 is known to regulate the genes involved in lipid and cholesterol production, such as *ACLY*, *ACACa*, and *FASN*, playing a key role in the induction of lipogenesis (Horton, Goldstein, and Brown 2002; Zhang et al. 2022; Figure 6B). In PRCC‐TFE3–inducible HK2 cells, ACLY, ACACa, FASN, and both transcriptional isoforms of SREBP1 (SREBP1a and SREBP1c) are all upregulated following Dox treatment (Figure 6B). These results suggest that PRCC‐TFE3–expressing cells enhance lipid synthesis pathways through the upregulation of SREBP1. Additionally, knockdown of HIF1α reduced the induction of SREBP1a and SREBP1c mRNA following Dox treatment in PRCC‐TFE3–inducible HK2 cells (Figures 6C and S7). These findings demonstrate that PRCC‐TFE3 regulates lipid synthesis pathways via the HIF1α/SREBP1 axis.

We next examined lipid accumulation in PRCC‐TFE3–expressing cells and kidneys. As expected, knockdown of PRCC‐TFE3 in UOK124 cells impaired lipid accumulation, as indicated by reduced LipiDye II fluorescence, whereas oleic acid treatment resulted in saturated LipiDye II fluorescence in these cells (Figures S7 and S9). Similarly, knockdown of either HIF1α or SREBP1 also reduced lipid accumulation in UOK124 cells (Figures 6E,F and S7). Furthermore, using adipophilin (ADFP) antibodies, we confirmed that *PRCC‐TFE3* KI kidneys exhibited stronger positive staining for lipid accumulation, whereas ADFP staining was reduced in *PRCC‐TFE3* KI/*Hif1α* KO kidneys (Figure 6G). These results underscore the enhanced lipid synthesis mediated by the PRCC‐TFE3/HIF1α/SREBP1 axis in TFE3‐RCC.

Finally, we investigated the roles of HIF1α and SREBP1 in TFE3‐RCC by performing anchorage‐independent growth analysis with PRCC‐TFE3, HIF1α, or SREBP1 knockdown in UOK124 cells. As expected, PRCC‐TFE3 knockdown resulted in reduced colony formation in 3D soft‐agar culture (Figure 3H). The proliferation in 2D dish culture remained unaffected in these cells (Figure S7). Similarly, knockdown of HIF1α or SREBP1 inhibited colony formation in 3D soft‐agar culture (Figure 6I). These data suggest that the PRCC‐TFE3/HIF1α/SREBP1 axis is critical for anchorage‐independent growth in TFE3‐RCC. The PRCC‐TFE3/HIF1α axis enhances glycolysis and TCA cycle flux. The increased glycolytic activity provides substrates for the TCA cycle, which are utilized for de novo lipid synthesis via SREBP1, which is upregulated by PRCC‐TFE3/HIF1α (Figure 6J). Overall, our data highlight the significance of the PRCC‐TFE3/HIF1α/SREBP1 axis in TFE3‐RCC development.

## 3 Discussion

Our study offers new insights into the molecular mechanisms of TFE3‐RCC, highlighting the pivotal role of the PRCC‐TFE3/HIF1α/SREBP1 axis in tumor development and progression. We found that HIF1α and HIF2α are direct transcriptional targets of PRCC‐TFE3 and confirmed their expression in TFE3‐RCC tissues, thereby establishing a clear connection between the TFE3 fusion protein and the hypoxia signaling pathway, which is central to many cancers, including ccRCC (Chappell, Payne, and Rathmell 2019; Wicks and Semenza 2022; Chen et al. 2023). In ccRCC, *VHL* inactivation leads to HIF accumulation and metabolic alterations (Linehan et al. 2019). Likewise, mutations in fumarate hydratase (*FH*) and succinate dehydrogenase (*SDH*) subunits, which are associated with hereditary leiomyomatosis and RCC (HLRCC) and hereditary paraganglioma–pheochromocytoma syndrome, respectively, also induce metabolic perturbations and HIF stabilization (Linehan et al. 2019). In these cases, the buildup of oncometabolites (fumarate or succinate) inhibits prolyl hydroxylases, thus stabilizing HIF even under normoxic conditions. This phenomenon, known as “pseudohypoxia,” drives metabolic reprogramming and tumorigenesis. However, in contrast to ccRCC, where HIF accumulation is primarily due to *VHL* inactivation (Foster et al. 1994; Gnarra et al. 1994; Mandriota et al. 2002; Semenza 2012b; Gossage, Eisen, and Maher 2015), or to *FH*‐ and *SDH*‐deficient RCC, where HIF is stabilized by oncometabolite accumulation, our findings indicate that HIF upregulation in TFE3‐RCC takes place at the transcriptional level, revealing a unique mode of HIF activation in this RCC subtype. Importantly, our in vivo studies using genetically engineered mouse models revealed that *Hif1α*, but not *Hif2α*, is essential for TFE3‐RCC development. This finding contrasts with some previous studies in ccRCC cell lines demonstrating a more prominent role for HIF2α in tumorigenicity (Kondo et al. 2003). However, our results align with recent studies using genetically engineered mice with mutated p53, which is not observed in humans, demonstrating the crucial role of *Hif1α* in ccRCC formation (Hoefflin et al. 2020). Our metabolic analyses revealed that the PRCC‐TFE3/HIF1α axis induces significant metabolic reprogramming in TFE3‐RCC cells. Specifically, our study showed that this axis upregulates both glycolysis and oxidative phosphorylation, thereby enhancing TCA cycle flux. This metabolic shift appears to be critical for supporting tumor growth and survival. The upregulation of glycolysis through HIF1α target genes (such as *HK2*, *GLUT1*, *PDK1*, and *LDHA*) provides cancer cells with rapid ATP production and biosynthetic intermediates (Kierans and Taylor 2021). At the same time, the sustained activity of the TCA cycle, particularly under low glutamine conditions, indicates a metabolic flexibility that may offer a growth advantage to TFE3‐RCC cells. A key finding of our study is the identification of SREBP1 as a downstream effector of the PRCC‐TFE3/HIF1α axis. SREBP1, a master regulator of lipid synthesis (Horton, Goldstein, and Brown 2002), is upregulated in response to PRCC‐TFE3 expression and HIF1α activation, leading to increased expression of lipogenic enzymes (ACLY, ACACa, and FASN) and enhanced lipid accumulation in TFE3‐RCC cells and tissues. Our data indicate that the PRCC‐TFE3/HIF1α/SREBP1 axis plays a pivotal role in this metabolic shift by concurrently suppressing ketogenesis and promoting lipid synthesis.

The inverse relationship between ketogenesis and tumor development in our mouse models provides further insight into the metabolic adaptations in TFE3‐RCC. The suppression of ketone body production, which is reversed by *Hif1α* knockout, suggests that acetyl‐CoA is preferentially channeled toward lipid synthesis rather than ketone body formation. This metabolic rewiring may be crucial for providing the building blocks necessary for rapid cell proliferation and membrane synthesis in cancer cells (DeBerardinis et al. 2008; Fu et al. 2021).

Our findings underscore the critical importance of the PRCC‐TFE3/HIF1α/SREBP1 axis in TFE3‐RCC progression, highlighting its potential as a therapeutic target. Given the critical role of HIF1α in TFE3‐RCC development, inhibiting HIF1α may be an effective therapeutic strategy for treating this cancer subtype. Metabolic intervention could be another therapeutic strategy by disrupting the glycolytic and/or lipogenic programs in TFE3‐RCC cells. Specifically, inhibitors targeting glycolytic enzymes or the SREBP1 pathway may serve as promising therapeutic candidates (Pusapati et al. 2016; Batchuluun, Pinkosky, and Steinberg 2022; Zhang et al. 2022; Zhao, Lin, and Wang 2022). The metabolic signatures identified in our study (e.g., increased lipid accumulation and decreased ketone bodies) could potentially serve as biomarkers for disease progression or treatment response in TFE3‐RCC patients.

Although our study provides significant insights into TFE3‐RCC biology, several questions remain to be addressed in future research. We demonstrated an increase in PRCC‐TFE3 protein levels and elevated target gene expression under low glutamine conditions. Clarifying how PRCC‐TFE3 is regulated by glutamine concentration will provide a better understanding of the molecular pathogenesis and offer a foundation for developing novel therapeutics for TFE3‐RCC. We have proven that HIF1α and HIF2α are directly regulated by PRCC‐TFE3. We then demonstrated that SREBP1 expression is regulated by both PRCC‐TFE3 and HIF1α. Our ChIP‐seq data show significant binding of HA‐PRCC‐TFE3 to the *SREBP1* gene body in HK2 cells (data not shown), suggesting direct regulation of SREBP1 by PRCC‐TFE3. Conversely, *PRCC‐TFE3* KI/*Hif1α* KO kidneys exhibited suppressed SREBP1 expression, and HIF1α knockdown reduced SREBP1 expression induced by PRCC‐TFE3 in HK2 cells. Together, these findings suggest that PRCC‐TFE3 and HIF1α co‐regulate SREBP1 expression. A detailed understanding of the molecular interactions within the PRCC‐TFE3/HIF1α/SREBP1 axis could lay the groundwork for innovative drug discovery.

Interestingly, we observed an increase in polyploid cells expressing PRCC‐TFE3 (Figure 3F). Polyploidy can arise from incomplete mitosis or disrupted checkpoints and is associated with both senescence and malignant transformation, often through genomic instability. Notably, ASPL‐TFE3 expression has been shown to induce cellular senescence (Ishiguro and Yoshida 2016), suggesting that TFE3 fusion proteins may broadly affect senescence‐related pathways. Although the precise mechanism remains unclear, PRCC‐TFE3 might similarly disrupt cell‐cycle regulation or checkpoints, leading to polyploid cell accumulation. Additionally, the potential involvement of HIF1α/HIF2α in this process cannot be ruled out, given their known roles in cell cycle regulation and genomic stability. Further research is needed to determine if this phenomenon represents senescence, genomic adaptation, or tumor progression, and to explore potential interactions between TFE3 fusion proteins and hypoxia‐inducible factors in these processes.

In conclusion, our study elucidates a novel PRCC‐TFE3/HIF1α/SREBP1 axis that drives metabolic reprogramming and tumor growth in TFE3‐RCC. These findings not only advance our understanding of TFE3‐RCC pathogenesis but also provide a rationale for exploring new therapeutic strategies targeting this aggressive form of kidney cancer. Future studies building on these insights may lead to improved treatments and outcomes for patients with TFE3‐RCC and potentially other nccRCC subtypes.

## 4 Experimental Procedures

### 4.1 Cell Lines

HEK293 cell lines, which express HA‐PRCC‐TFE3 in a Dox‐dependent manner, were established using the Flp‐In T‐Rex System (Thermo Fisher Scientific) as previously described (Baba et al. 2006) and cultured in DMEM with 10% tetracycline‐free fetal bovine serum (FBS) and selection antibiotics, 15 μg/mL blasticidin S and 150 μg/mL hygromycin B. The UOK124 human RCC cell line from TFE3‐RCC was established as previously described (Shipley et al. 1995) and cultured in DMEM with 10% FBS and penicillin–streptomycin (100 U/mL). HK‐2 cell lines, which express HA‐PRCC‐TFE3 in a Dox‐dependent manner, were established using the Tet‐On 3G‐Inducible Expression System and cultured in advanced DMEM/F‐12 with 1.5% tetracycline‐free FBS and selection antibiotics, 2 μg/mL blasticidin S and 0.8 μg/mL puromycin. For gene expression profiling experiments, HEK293 cell lines or HK‐2 cell lines were cultured with or without 250 ng/mL Dox.

Knockdown of PRCC‐TFE3 was achieved using the miRE–based RNAi system or pLKO.1‐puro (SHC0‐02, Sigma) as reported previously (Funasaki et al. 2022). The shRNA‐miRE sequences targeting respective genes were inserted into SGEP plasmid vectors (Addgene #111170). For the experiment using the green fluorescence channel, SGEP plasmid vectors without EGFP sequence were used. The TFE3‐RCC cell lines were transduced with the viral supernatants containing integrated shRNA vectors and subsequently selected with puromycin (5 μg/mL, Promega). The following sequences were used to target the respective genes:

shTFE3#1, 5′‐TCAGATAAACAAATGAGGGGGT‐3′;

shTFE3#3, 5′‐TATTATTTTAATCACAAACCTA‐3′;

shHIF1A, 5′‐TTTTCTACCAATTCCAACTTGA‐3′.

For the pLKO clones (Sigma), following sequences were used:

shHIF1A‐#8, 5′‐CCGCTGGAGACACAATCATAT‐3′;

shHIF1A‐#10, 5′‐GTGATGAAAGAATTACCGAAT‐3′;

shSREBP1‐#5, 5′‐GCCATCGACTACATTCGCTTT‐3′;

shSREBP1‐#7, 5′‐CCAGAAACTCAAGCAGGAGAA‐3′.

shTFE3#1 and shTFE3#3 were designed to target Exon 10 of TFE3, which knocks down both PRCC‐TFE3 and wild‐type TFE3.

### 4.2 Genetically Engineered Mouse Model

The TFE3‐RCC mouse model, *Rosa26‐lSl‐PRCC‐TFE3* KI, and *Cadherin 16‐Cre* (Ksp‐Cre) mice (Baba et al. 2019) were crossed with *Hif1α* ^*f/f*^ (*Hif1α* KO; Ryan, Lo, and Johnson 1998), *Hif2α* ^*f/f*^ (*Hif2α* KO) (Gruber et al. 2007), and double *Hif1α* ^*f/f*^ and *Hif2α* ^*f/f*^ (*Hif1α*/*Hif2α* DKO) mice. The mice were housed at the Center for Animal Resources and Development (CARD) at Kumamoto University and euthanized by cervical dislocation after isoflurane inhalation anesthesia, following the guidelines of the Kumamoto University Animal Care and Use Committee.

### 4.3 Immunohistochemistry

The resected tissues were fixed in 10% formalin and embedded in paraffin. Paraffin‐embedded samples were sectioned into 4‐μm slices for histological analysis. Hematoxylin and eosin (H&E) staining was performed using standard procedures. For immunohistochemistry, antigen retrieval was conducted in an autoclave at 121°C for 15 min using 0.01 M citrate buffer (pH 7.0). To block endogenous peroxidase activity, the sections were treated with 3% H_2_O_2_ and incubated in Tris‐buffered saline with 0.1% Tween‐20 (TBS‐T) containing 5% goat serum to block nonspecific binding. The following primary antibodies were used: mouse monoclonal anti‐HIF1α (Enzo Life Sciences, ADI‐OSA‐602; 1:50), rabbit polyclonal anti‐HIF1α (Novus Biologicals, NB100‐449; 1:100), rabbit polyclonal anti‐HIF2α (Novus Biologicals, NB100‐122; 1:100), rabbit polyclonal anti‐CD31 (Proteintech, 28083‐1‐AP; 1:1000), and rabbit polyclonal anti‐ADFP (Proteintech, 15294‐1‐AP; 1:2000). The secondary antibodies used were horseradish peroxidase–conjugated goat anti‐rabbit IgG (Nichirei, 424141) and goat anti‐mouse IgG (Nichirei, 414171), with visualization achieved using the Liquid DAB+ Substrate Chromogen System (DAKO, K3468). Nuclei were counterstained with hematoxylin, and the slides were imaged using a BZ‐X700 microscope (Keyence).

### 4.4 RNA‐Seq

Total RNA was extracted using TRIzol reagent, and its quality was assessed using a BioAnalyzer 2100. Samples with an RNA integrity number (RIN) greater than 8 were selected for further processing. Library preparation was performed using the TruSeq Stranded mRNA Library Prep kit, following the manufacturer's protocol. Sequencing was conducted on an Illumina NextSeq 500 platform using the NextSeq 500/550 High Output v2.5 Kit, generating single‐end reads of 75 nucleotides in length. The resulting sequencing reads underwent initial processing using Trim Galore! (Version 0.6.6, incorporating cutadapt Version 2.5) to remove adapter sequences and trim low‐quality ends. The trimmed reads were subsequently aligned to the human genome (UCSC hg38 assembly) using STAR (Version 2.7.9a). Transcript quantification and differential expression analysis were performed using RSEM (Version 1.3.3) for transcripts per million (TPM) calculations and DESeq2 (Version 1.42.1) for statistical analysis of differential expression. The gene transfer format (GTF) file used for annotation was obtained from the UCSC hg38 or GRCm38 genome build.

### 4.5 DNA Microarray Analysis

Total RNA was extracted from HEK293 cell lines expressing HA‐PRCC‐TFE3 in a Dox‐dependent manner using TRIzol reagent (Invitrogen). cDNA preparation and probe array hybridization were performed according to the manufacturer's instructions (Affymetrix), using Affymetrix GeneChip HG‐U133 Plus 2.0 arrays. Data normalization (RMA–based), as well as PCA and ANOVA, were conducted using Partek Genomics Suite 6.6.

### 4.6 Quantitative RT‐PCR (RT‐qPCR)

Total RNA was isolated using TRIzol Reagent (Thermo Fisher Scientific) and reverse transcribed to cDNA using ReverTra Ace qPCR RT Master Mix (Toyobo, Osaka, Japan). qPCR assays were performed with the Roche LightCycler 96 system (Roche, Mannheim, Germany) and the Thunderbird SYBR qPCR Mix (Toyobo). Each value was normalized using the value of Rps18 as an internal control. Primer sequences are listed as follows:

HIF1α For: 5′‐TATGAGCCAGAAGAACTTTTAGGC‐3′;

HIF1α Rev: 5′‐CACCTCTTTTGGCAAGCATCCTG‐3′;

HIF2α For: 5′‐AGCTTCCTGCGAACACACAA‐3′;

HIF2α Rev: 5′‐TCACCACGGCAATGAAACC‐3′;

HK2 For: 5′‐GAAGATGCTGCCCACCTTTG‐3′;

HK2 Rev: 5′‐CACCCAAAGCACACGGAAG‐3′;

GLUT1 For: 5′‐CCAGAAGGTGATCGAGGAGTT‐3′;

GLUT1 Rev: 5′‐AAAAGGCCCACAGAGAAGGAG‐3′;

PDK1 For: 5′‐AGATGAGTGACCGAGGAGGTG‐3′;

PDK1 Rev: 5′‐CCATAACCAAAACCAGCCAGA‐3′;

LDHA For: 5′‐CAGCCCGATTCCGTTACCTA‐3′;

LDHA Rev: 5′‐ACACCAGCAACATTCATTCCAC‐3′;

SREBP1a For: 5′‐GGAGGGGTAGGGCCAACGGCCT‐3′;

SREBP1a Rev: 5′‐CATGTCTTCGAAAGTGCAATCC‐3′;

SREBP1c For: 5′‐TCAGCGAGGCGGCTTTGGAGCAG‐3′;

SREBP1c Rev: 5′‐CATGTCTTCGATGTCGGTCAG‐3′;

ACLY For: 5′‐GCCCATCCCCAACCAGCCAC‐3′;

ACLY Rev: 5′‐TTGCAGGCGCCACCTCATCG‐3′;

ACACa For: 5′‐CGGAAGGGACAGTAGAAATCA‐3′;

ACACa Rev: 5′‐AGTCGCTCAGCCAAGTGGA‐3′;

FASN For: 5′‐CACAGGGACAACCTGGAGTT‐3′;

FASN Rev: 5′‐ACTCCACAGGTGGGAACAAG‐3′;

GPNMB For: 5′‐CATGGCCGAAAGCTCCCTA‐3′;

GPNMB Rev.: 5′‐GGTGGGGTCAGAAATGATGG‐3′;

RPS18 For: 5′‐TTGCGAGTACTCAACACCAACA‐3′;

RPS18 Rev: 5′‐TCTGCTTTCCTCAACACCACA‐3′.

### 4.7 ChIP‐Seq and Quantitative PCR (ChIP‐qPCR) Analysis

HK2 cell lines, which express HA‐PRCC‐TFE3 in a Dox‐dependent manner, were cultured with Dox for 24 h and then crosslinked with 1% formaldehyde at room temperature for 5 min, followed by incubation with 125 mM glycine. Nuclei preparation, chromatin digestion with micrococcal nuclease, ChIP with anti‐HA antibody (3F10, Roche), and DNA purification were performed using SimpleChIP Plus Enzymatic Chromatin IP Kit (Magnetic Beads; #9005, Cell Signaling, Danvers, MA, USA) according to the manufacturer's protocol. DNA libraries required for high‐throughput sequencing were prepared from the post‐ChIP DNA using the NEBNext Ultra DNA Library Prep Kit and NEBNext Multiplex Oligos for Illumina (New England BioLabs). The multiplexed libraries were clustered and sequenced using the NextSeq 500 Kit (75 cycles) and the NextSeq Desktop Sequencing System (Illumina). Peak detection was performed using the MACS algorithm (PMID:18798982) in the Strand NGS software (Strand Life Sciences). HA‐TFE3 binding was identified by significant enrichment of each signal over input DNA peaks with a *p*‐cutoff value of 10^−5^. Visualization of ChIP‐seq data was performed using the Strand NGS software. In ChIP qPCR, DNA purification was performed in the same manner as in ChIP seq. The primer sequences utilized in the qPCR assays are listed as follows:

HIF1α M‐boxes For: 5′‐TTCCTCCGCCGCTAAACA‐3′;

HIF1α M‐boxes Rev: 5′‐CGCTCTCAGCCAATCAGGAG‐3′;

HIF2α M‐box1 For: 5′‐AGGTGCTCGGCGTCTGAAC‐3′;

HIF2α M‐box1 Rev: 5′‐ACGCCCGCTTACCTTTTCTTC‐3′;

HIF2α M‐box2 For: 5′‐TACAATCCTCGGCAGTGTCC‐3′;

HIF2α M‐box2 Rev: 5′‐AAAAAGTGGCTCGAATGCTG‐3′;

HIF2α M‐box3 For: 5′‐CGCAGCCCTAGATCAATTTCCTT‐3′;

HIF2α M‐box3 Rev: 5′‐GGCGATGCGCCCTTTTC‐3′.

### 4.8 Luciferase Reporter Gene Assay

A 933‐bp fragment of the 5′ region of the human HIF1α gene (human BAC clone: PR11‐243C17) and a 1049‐bp fragment of the human HIF2α gene (PR11‐593E8), containing the TFE3 binding peak, were amplified by PCR using KOD‐Plus‐Neo polymerase (Toyobo Co.). The PCR products, amplified with promoter‐specific primers (HIF1α_For: 5′‐ATGTACATGCTAGCCATCTGAGCAACGAGACCAA‐3′; HIF1α_Rev: 5′‐AACTTGATAAGCTTCGAGGGAATGGGCTTACTTT‐3′; HIF2α_For: 5′‐ATGTACATGCTAGCATCACACTGGGGAACCAGAC‐3′; HIF2α_Rev: 5′‐AACTTGATAAGCTTGCTGTCAGACCCGAAAAGAG‐3′), were digested with *Nhe*I and *Hin*dIII and ligated into the pGL4‐Basic Vector (Promega). The reporter plasmid vector was co‐transfected with a control vector (phRL) into PRCC‐TFE3‐inducible HEK293 cells. Luciferase activity was measured after 24 h of treatment with or without Dox, using the Dual‐Luciferase Reporter Assay System according to the manufacturer's protocols (Promega).

### 4.9 Immunofluorescence

UOK124 cells were cultured on coverslips with the specified concentrations of glutamine for 24 h. The cells were then fixed with 4% formaldehyde for 5 min at 37°C, washed with PBS, and blocked with FBS before permeabilization with 0.5% Triton X‐100 containing 1% FBS. Following additional washes, the cells were incubated overnight at 4°C with an anti‐TFE3 antibody (Sigma, HPA023881). After washing, the cells were stained with an anti‐rabbit Alexa Fluor 488 antibody (Thermo Fisher). Following three washes, the cells were incubated with DAPI (Dojindo) for 5 min, and fluorescence images were captured using a BZ‐X700 microscope. (Keyence).

### 4.10 ATP Quantification Analysis

UOK124 cells were seeded at a density of 5000 cells per well, in quadruplicate, on a 96‐well plate and cultured for 24 h. After incubation, the cells were lysed and mixed for 15 min according to the manufacturer's protocol using the CellTiter‐Glo 2.0 Cell Viability Assay (Promega, G9241). Luminescence was then measured with a Synergy H1 plate reader (BioTek).

### 4.11 Glucose Uptake Assay

UOK124 cells were washed twice with PBS, harvested using Accutase (Nacalai, 12679‐54), and then washed once more with PBS. The cells (1 × 10^6^ cells/mL) were incubated for 2 h in a 37°C water bath with a medium containing 100 μM 2‐NBDG (Peptide Institute, 23002‐v). After washing once with PBS, the cells were resuspended in PBS containing propidium iodide (5 μg/mL) and analyzed by BD FACSCanto II (BD Biosciences).

### 4.12 α‐Ketoglutarate Quantitation

Cellular α‐Ketoglutarate concentration was measured using the α‐Ketoglutarate Quantitation Kit (Sigma, AK541‐1KT) according to the manufacturer's protocol.

Briefly, 2 × 10^6^ cells were washed with PBS and harvested in a 100‐μL ice‐cold α‐KG buffer. The lysates were then centrifuged at 13,000*g* for 10 min, and the supernatant was mixed with α‐KG converting enzyme, α‐KG development enzyme mix, and fluorescent peroxidase substrate in α‐KG assay buffer, then incubated for 30 min at 37°C. The fluorescence intensity (*λ*_ex_ = 535/*λ*_em_ = 587 nm) was quantified using a Synergy H1 plate reader and normalized to the α‐KG standard reaction.

### 4.13 Extracellular Flux Analysis

OCR and ECAR were measured using a Seahorse XF HS Mini Analyzer (Seahorse Bioscience) with the Agilent Seahorse XF Cell Mito Stress Test Kit (Agilent Technologies), according to the manufacturer's protocol. Briefly, cells were seeded at a density of 1 × 10^4^ cells/well in triplicate in growth medium and incubated for 24 h. The cells were then washed and incubated with non‐buffered DMEM (XF DMEM medium, pH 7.4; Agilent Technologies, 103575‐100) supplemented with 10 mM glucose (Agilent Technologies, 103577‐100), 1 mM pyruvate (Agilent Technologies, 103578‐100), and 2 mM l‐glutamine (Agilent Technologies, 103579‐100) for 1 h at 37°C, in a CO_2_‐free incubator. The XF well plate was then loaded into the cartridge and analyzed using the Seahorse XF HS Mini Analyzer, with sequential addition of oligomycin (10 μM), FCCP (10 μM), and rotenone and antimycin A (5 μM for each).

### 4.14 Gas Chromatography‐Mass Spectrometry Analysis

Frozen mouse kidney tissue samples were plunged into Milli‐Q water/methanol/chloroform (at a ratio of 1:2.5:1) containing internal standards (isopropylmalic acid). The samples were homogenized at 2300 r.p.m. for 30 s with a bead beater‐type homogenizer (Beads Crusher μT‐12, Titec). The homogenized sample was shaken at 1200°r.p.m. and 37°C for 30 min and centrifuged at 16,000*g* and 4°C for 3 min. Subsequently, 225 μL of the upper aqueous layer was transferred to a new tube. The remaining sample was mixed with 200 μL Milli‐Q water and centrifuged again at 16,000 g and 4°C for 3 min. The upper water layer was mixed with the aqueous layer. The solution was evaporated using a centrifugal evaporator (DNA120OP230, Thermo Fisher Scientific) and lyophilized (FD‐1‐84A, FST). Samples were analyzed on a Shimadzu TQ8050GC‐MS/MS. The chromatograms and mass spectra were analyzed utilizing the GC‐MS solution software v4.5 (Shimadzu). Compounds were determined with the Smart Metabolite Database v2 (Shimadzu).

### 4.15 Cell Proliferation Assay

UOK124 cells expressing shCont. or shTFE3 were cultured in 24‐well plates for the indicated durations. Resazurin solution (DPBS, pH 7.5; resazurin sodium salt, Wako 191‐07581) was added to the media to a final concentration of 0.03 mg/mL, followed by incubation for an additional 2–4 h at 37°C in 5% CO₂. The cultured media were then collected, and fluorescence was quantified with excitation at 560 nm and emission at 590 nm.

### 4.16 FACS Lipid Analysis

UOK124 cells cultured for 24 h were washed once with PBS and then incubated with phenol red‐free DMEM containing 0.1 μM LipiDye II (FDV‐0027, Funakoshi Co. Ltd.) for 30 min. Oleic acid (200 μM) was added 24 h prior to the incubation with LipiDye II as a lipid‐saturated positive control. The cells were then detached with Accutase, washed with PBS once, and analyzed in PBS by BD FACSCanto II.

### 4.17 Anchorage‐Independent Tumor Cell Growth Assay

The anchorage‐independent tumor cell growth assay was conducted as previously described (Kodama et al. 2020). Briefly, a single‐cell suspension of UOK124 cells expressing shCont., shSREBP1, or shHIF1α (1 × 10^4^ cells) was prepared in DMEM supplemented with 10% FCS and 0.33% agar. This suspension was then layered onto a base of the same media containing 0.5% agar in a six‐well plate. After 21 days of culture, colonies were stained with 2‐(4‐iodophenyl)‐3‐(4‐nitrophenyl)‐5‐phenyl‐2H‐tetrazolium chloride (INT, Dojindo). The percentage of colony formation was quantified using ImageJ software.

### 4.18 Statistical Analysis

All statistical analyses were performed using GraphPad Prism 10 (GraphPad software). Detailed information about the statistical methods for each experiment can be found in the figure legends and the Section 4.

## Author Contributions

**Hidekazu Nishizawa:** investigation, validation, writing – original draft, visualization. **Shintaro Funasaki:** conceptualization, formal analysis, funding acquisition, visualization, investigation, writing – original draft. **Wenjuan Ma:** conceptualization, data curation, formal analysis, funding acquisition, investigation, visualization, writing – original draft. **Yoshiaki Kubota:** resources. **Kazuhide Watanabe:** data curation, formal analysis. **Yuichiro Arima:** investigation, project administration. **Shoichiro Kuroda:** validation. **Takaaki Ito:** investigation. **Mitsuko Furuya:** investigation. **Takanobu Motoshima:** investigation, funding acquisition. **Akira Nishiyama:** investigation. **Sally Mehanna:** validation. **Yorifumi Satou:** formal analysis. **Hisashi Hasumi:** conceptualization. **Ryosuke Jikuya:** conceptualization, resources. **Kazuhide Makiyama:** project administration. **Tomohiko Tamura:** project administration. **Yuichi Oike:** project administration, resources. **Yasuhito Tanaka:** funding acquisition, project administration. **Toshio Suda:** funding acquisition, project administration. **Laura S. Schmidt:** resources. **W. Marston Linehan:** resources. **Masaya Baba:** conceptualization, formal analysis, funding acquisition, project administration, supervision, visualization, writing – review and editing, writing – original draft. **Tomomi Kamba:** funding acquisition, project administration.

## Ethics Statement

The Institutional Animal Care and Use Committee of Kumamoto University approved the experiments in accordance with international and national guidelines (Approval Number: A 2022‐114; Approval Date: December 12, 2022).

## Conflicts of Interest

The authors declare no conflicts of interest.

## Final Approval

All authors have reviewed the manuscript, approved its content, and agreed to its submission to the journal.

## Declaration of Generative AI in the Writing Process

Perplexity and ChatGPT 4o were utilized to enhance the manuscript's English language quality, focusing on sentence structure, word choice, and fluency. The AI was used exclusively for language refinement across all sections of the manuscript, including the abstract, introduction, results, discussion, and methods, with no involvement in the scientific content or analysis. All scientific aspects remain the original work of the authors, who thoroughly reviewed and edited the final manuscript to ensure its accuracy and authenticity.

## Supporting information


Figure S1.

Figure S2.

Figure S3.

Figure S4.

Figure S5.

Figure S6.

Figure S7.

Figure S8.

Figure S9.

## Data Availability

The original data presented in this study are publicly available in Gene Expression Omnibus with the Accession Numbers GSE278984, GSE281146, GSE283743, and GSE281281.
