# Supplementary material for: HIF1α Plays a Crucial Role in the Development of TFE3–Rearranged Renal Cell Carcinoma by Orchestrating a Metabolic Shift Toward Fatty Acid Synthesis
Source: Genes Cells. 2025 Jan 14;30(1):e13195. doi: 10.1111/gtc.13195 (PMC11729263; doi:10.1111/gtc.13195)
Supplement: Supplementary file 1 — Figure S1. [file GTC-30-0-s002.pdf]

Hallmark GSEA analysis of PRCC-TFE3 inducible cells, related to Figure 1

A

NAME	SIZE	NES	FDR q-val	NOM p-val
HALLMARK_HYPOXIA	189	1.879	0.007	0.000
HALLMARK_MTORC1_SIGNALING	194	1.519	0.087	0.000
HALLMARK_PROTEIN_SECRETION	95	1.461	0.093	0.018
HALLMARK_GLYCOLYSIS	195	1.448	0.079	0.002
HALLMARK_PI3K_AKT_MTOR_SIGNALI	103	1.340	0.160	0.053
HALLMARK_HEME_METABOLISM	191	1.288	0.201	0.043
HALLMARK_UV_RESPONSE_UP	151	1.278	0.185	0.054
HALLMARK_P53_PATHWAY	190	1.218	0.255	0.089

B

NAME	SIZE	NES	FDR q-val	NOM p-val
HALLMARK_HYPOXIA	57	1.686	0.105	0.002
HALLMARK_TNFA_SIGNALING_VIA_NFKB	55	1.612	0.106	0.007
HALLMARK_INTERFERON_ALPHA_RESPONSE	33	1.580	0.088	0.021
HALLMARK_UV_RESPONSE_UP	33	1.409	0.219	0.073

(A) Hallmark GSEA analysis of PRCC-TFE3 inducible HK2 cells treated with or without Dox for 24 hours. Only pathways with a NES greater than 1.2 and a q-value less than 0.2 are included.

(B) Hallmark GSEA analysis of PRCC-TFE3 inducible HEK293 cells treated with or without Dox for 24 hours. Only pathways with a NES greater than 1.2 and a q-value less than 0.25 are included.

Fig. S1
