# Supplementary material for: HIF1α Plays a Crucial Role in the Development of TFE3–Rearranged Renal Cell Carcinoma by Orchestrating a Metabolic Shift Toward Fatty Acid Synthesis
Source: Genes Cells. 2025 Jan 14;30(1):e13195. doi: 10.1111/gtc.13195 (PMC11729263; doi:10.1111/gtc.13195)
Supplement: Supplementary file 2 — Figure S2. [file GTC-30-0-s008.pdf]

Heat Map Depicting GSEA (Hypoxia Signature) of Differentially Expressed Genes in PRCC-TFE3–Expressing (Dox+) versus Control (Dox–) HK2 and 293 Cells

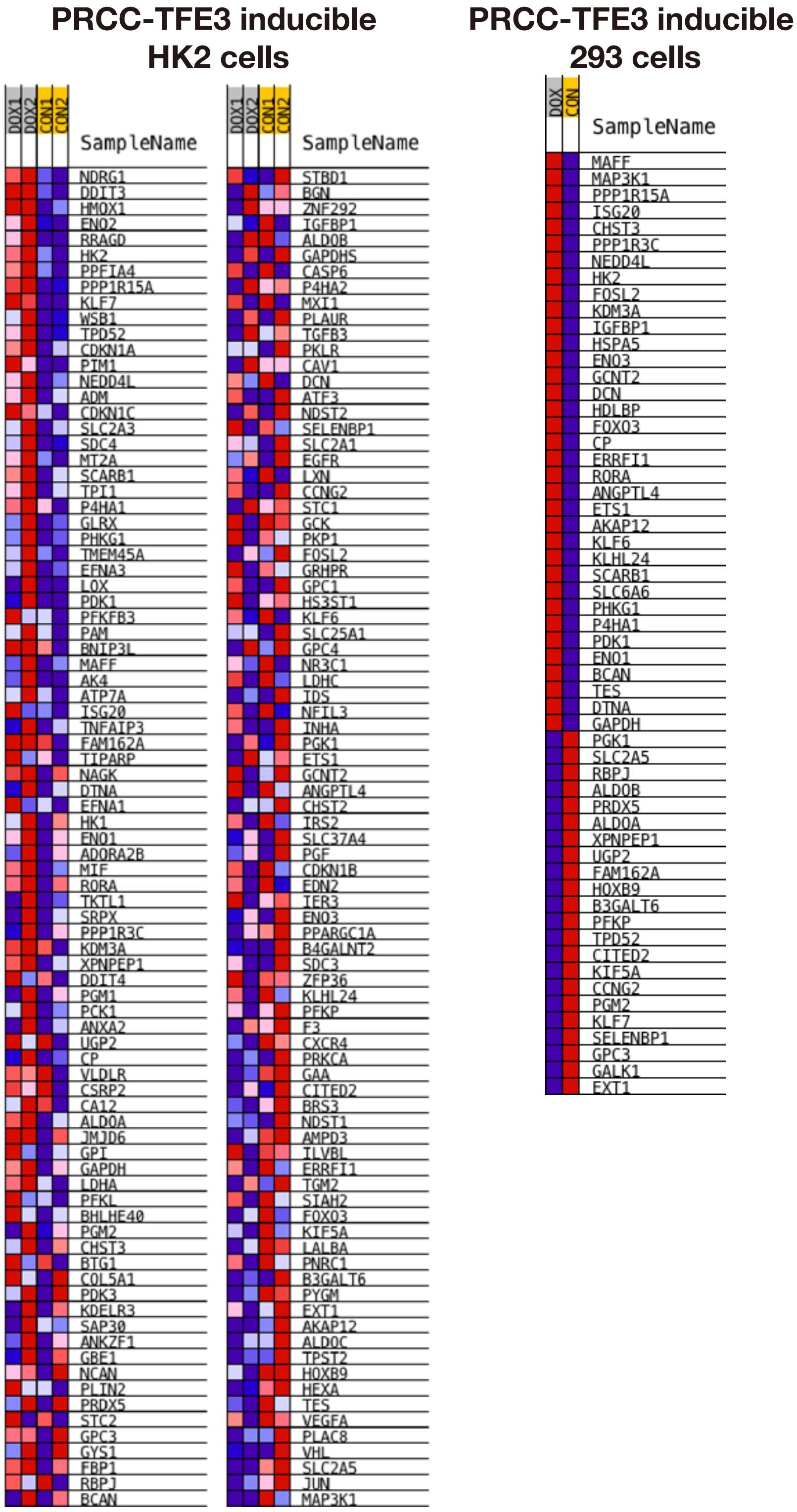

This heat map shows the Gene Set Enrichment Analysis (GSEA) results for hypoxia-related gene signatures, comparing PRCC-TFE3–expressing (Dox+) and control (Dox–) HK2 and 293 cells.

Fig. S2
