# Supplementary material for: HIF1α Plays a Crucial Role in the Development of TFE3–Rearranged Renal Cell Carcinoma by Orchestrating a Metabolic Shift Toward Fatty Acid Synthesis
Source: Genes Cells. 2025 Jan 14;30(1):e13195. doi: 10.1111/gtc.13195 (PMC11729263; doi:10.1111/gtc.13195)
Supplement: Supplementary file 3 — Figure S3. [file GTC-30-0-s001.pdf]

Upregulation of HIF1α-specific and HIF2α-Specific target genes in HK2 and HEK293 cells following PRCC-TFE3 Induction

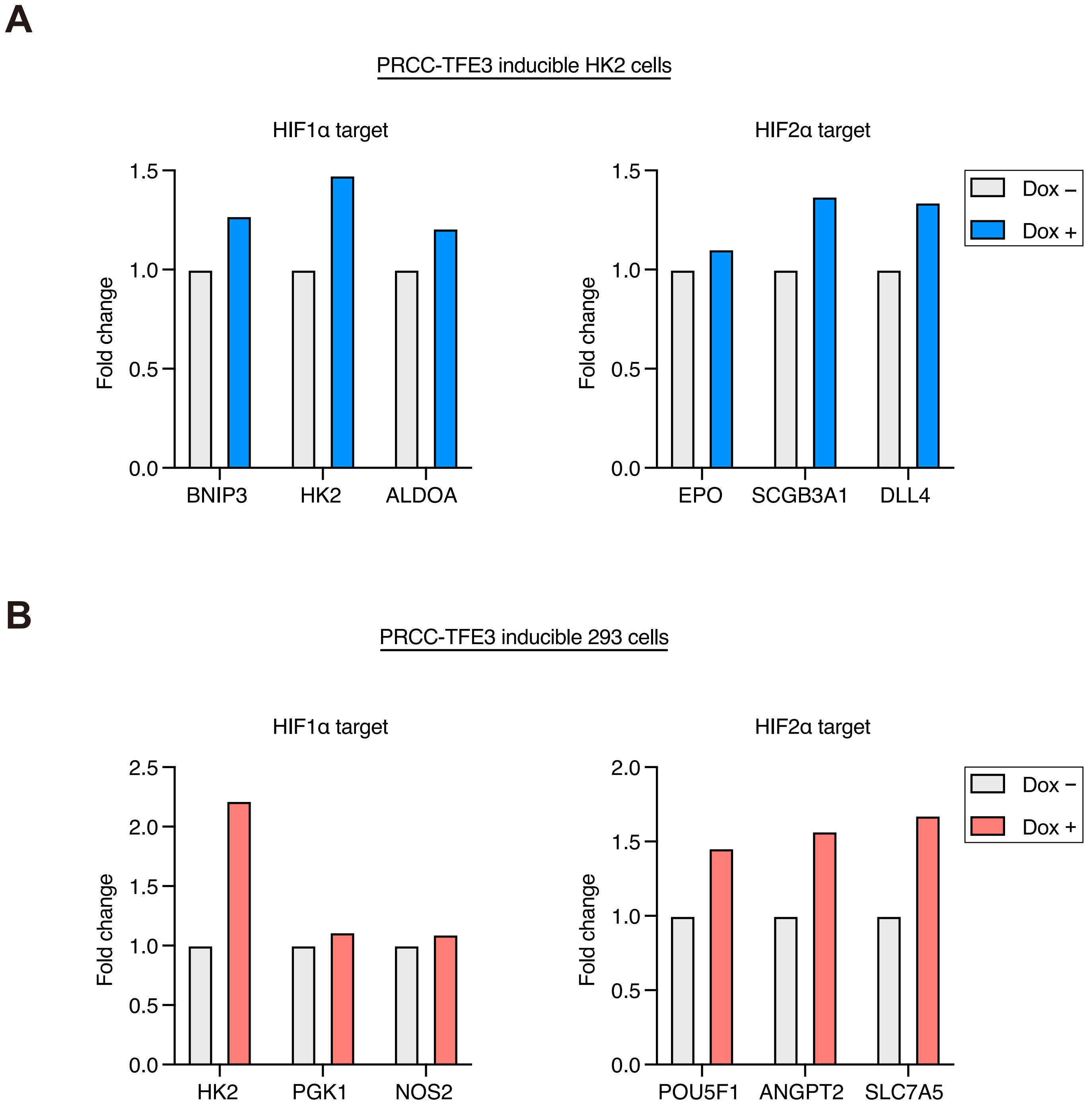

Single-sample RNA-seq (HK2) and microarray (HEK293) analyses reveal upregulation of certain HIF1α- and HIF2α-specific target genes following PRCC-TFE3 induction. Cells were treated with or without doxycycline (Dox), with Dox+ samples showing higher expression compared to Dox– controls.

Fig. S3
