# Supplementary material for: HIF1α Plays a Crucial Role in the Development of TFE3–Rearranged Renal Cell Carcinoma by Orchestrating a Metabolic Shift Toward Fatty Acid Synthesis
Source: Genes Cells. 2025 Jan 14;30(1):e13195. doi: 10.1111/gtc.13195 (PMC11729263; doi:10.1111/gtc.13195)
Supplement: Supplementary file 4 — Figure S4. [file GTC-30-0-s006.pdf]

Heat Map Depicting GSEA (Hypoxia Signature) of Differentially Expressed Genes in PRCC-TFE3–Expressing (Cre+) versus Control (Cre–) mouse kidneys.

PRCC-TFE3 KI
mouse kedneys

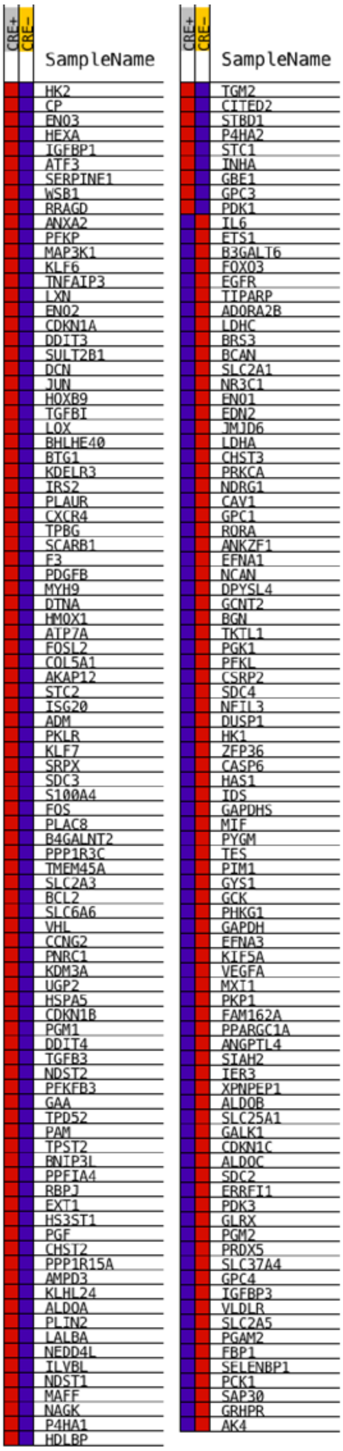

This heat map shows the Gene Set Enrichment Analysis (GSEA) results for hypoxia-related gene signatures, comparing PRCC-TFE3–Expressing (Cre+) and Control (Cre–) mouse kidneys.

Fig. S4
