# Supplementary material for: HIF1α Plays a Crucial Role in the Development of TFE3–Rearranged Renal Cell Carcinoma by Orchestrating a Metabolic Shift Toward Fatty Acid Synthesis
Source: Genes Cells. 2025 Jan 14;30(1):e13195. doi: 10.1111/gtc.13195 (PMC11729263; doi:10.1111/gtc.13195)
Supplement: Supplementary file 5 — Figure S5. [file GTC-30-0-s003.pdf]

Effects of glutamine and glucose concentrations on PRCC-TFE3 transcriptional activity, related to Figure 3

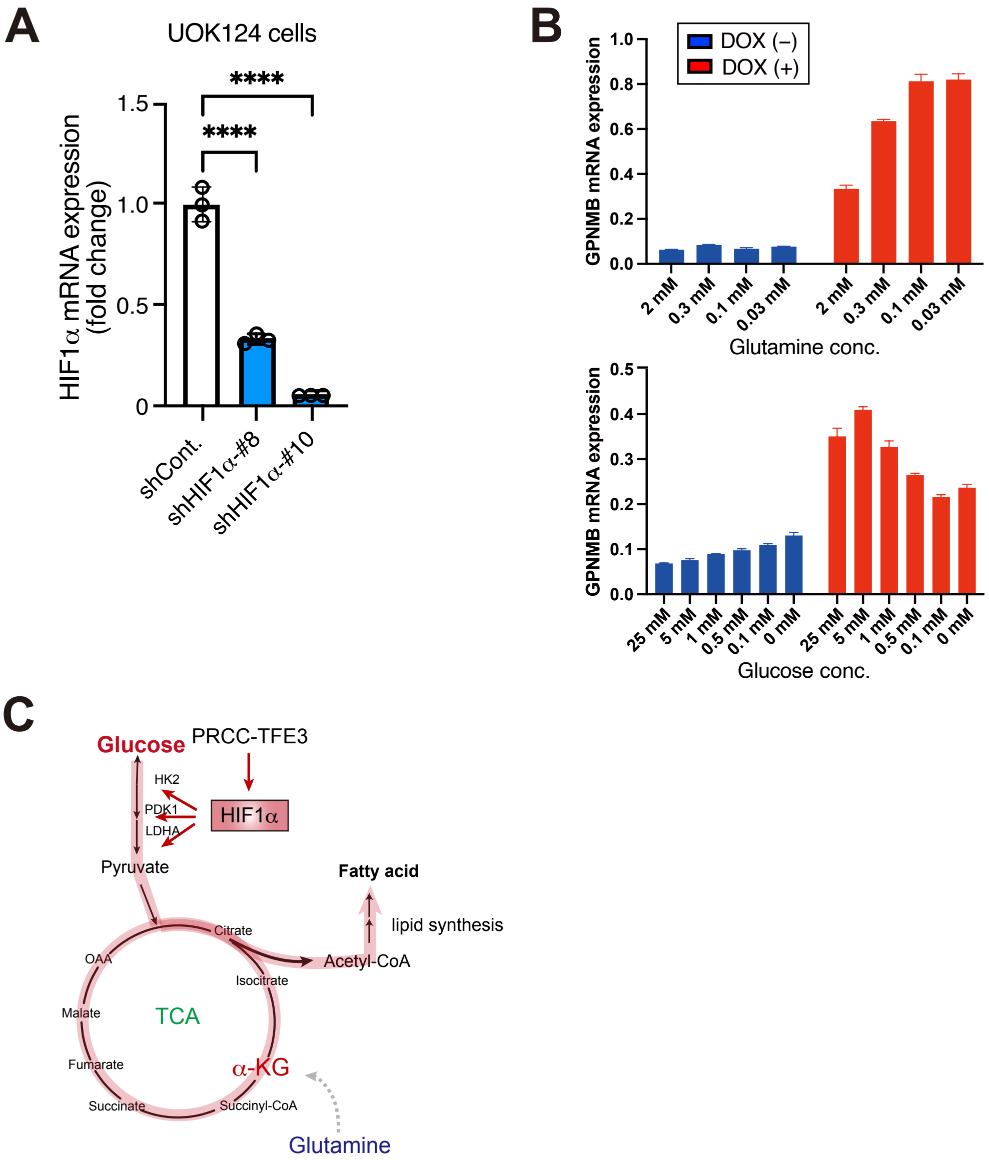

(A) RT-qPCR analysis of *HIF1α* in the patient-derived PRCC-TFE3 RCC cell line (UOK124) with PRCC-TFE3 knockdown (n = 3). Data are means ± SD. ****p < 0.0001 (Welch's t-test)
(B) PRCC-TFE3 inducible HK2 cells were treated with or without Dox for 24 hours in media containing varying concentrations of glutamine (upper) or glucose (lower).
RT-qPCR analysis was performed to assess PRCC-TFE3 transcriptional activity by measuring GPNMB mRNA expression. (n=3)
(C) Schematic figure illustrating glucose and glutamine metabolism.

Fig. S5
