# Supplementary material for: HIF1α Plays a Crucial Role in the Development of TFE3–Rearranged Renal Cell Carcinoma by Orchestrating a Metabolic Shift Toward Fatty Acid Synthesis
Source: Genes Cells. 2025 Jan 14;30(1):e13195. doi: 10.1111/gtc.13195 (PMC11729263; doi:10.1111/gtc.13195)
Supplement: Supplementary file 6 — Figure S6. [file GTC-30-0-s009.pdf]

Metabolome analysis of kidney tissues from the TFE3-RCC mouse model, related to Figure 5

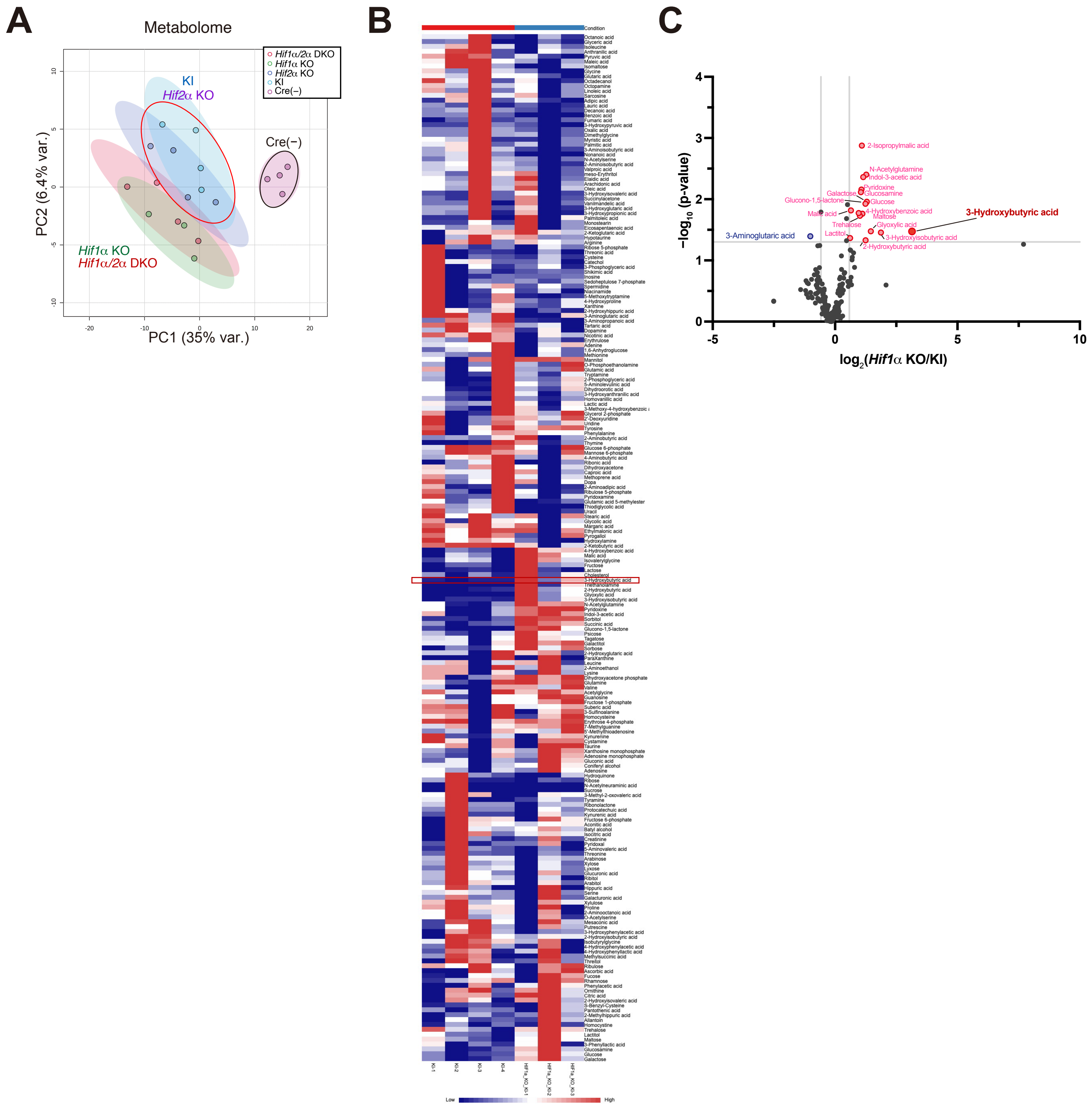

(A) PCA analysis of metabolite expression data

(B) Heatmap showing the expression levels of all metabolites in *PRCC-TFE3* KI and *PRCC-TFE3* KI / *Hif1α* KO samples.

(C) Volcano plots depicting metabolites significantly upregulated or downregulated by *Hif1α* knockout in *PRCC-TFE3* KI mouse kidneys.

The gray border lines indicate $p < 0.05$ or |log2 fold change| < 0.58 for significant changes.

Fig. S6
