# Supplementary material for: HIF1α Plays a Crucial Role in the Development of TFE3–Rearranged Renal Cell Carcinoma by Orchestrating a Metabolic Shift Toward Fatty Acid Synthesis
Source: Genes Cells. 2025 Jan 14;30(1):e13195. doi: 10.1111/gtc.13195 (PMC11729263; doi:10.1111/gtc.13195)
Supplement: Supplementary file 7 — Figure S7. [file GTC-30-0-s007.pdf]

# Regulation of lipid synthesis and tumor cell growth, related to Figure 6

**A**

Top regulated genes from ketone metabolic process (GO term)

Prkag2, AMP-activated protein kinase g2
Adipoq, related to lipid metabolism
Agt, angiotensinogen
Erg1, induced by PPAR alpha and PPAR gamma coactivation
Fabp5, related to lipid metabolism, active Akt pathway and promote cancer cell growth
Pparg, related to lipid metabolism
Srebf1, related to lipid metabolism, regulated by mTOR
Appl2, metabolic factor for cell growth and insulin-stimulated glucose uptake
Wnt4, Wnt family member 4
Insig1, related to lipid metabolism, insulin-induced gene1
Abcd2, related to lipid metabolism, ATP binding casette subfamily D member 2
Irs2, related to lipid metabolism, insulin receptor substrate 2
Ugt1a1, uridine diphosphate glucuronosyl transferase 1A1
Dkk3, enhance insulin sensitivity and glucose tolerance
Insig2, related to lipid metabolism, insulin-induced gene 2
Hsd17b1, steroidogenic enzymes

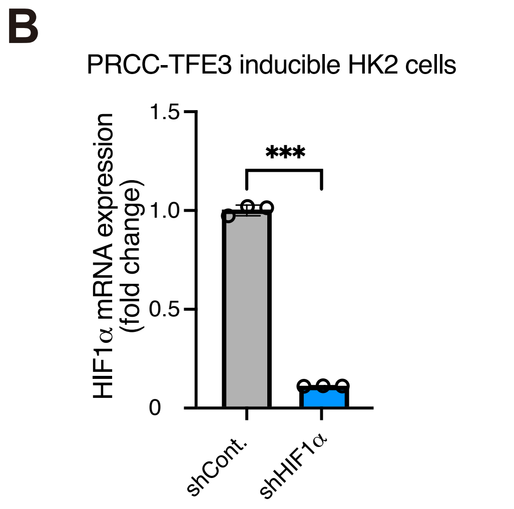


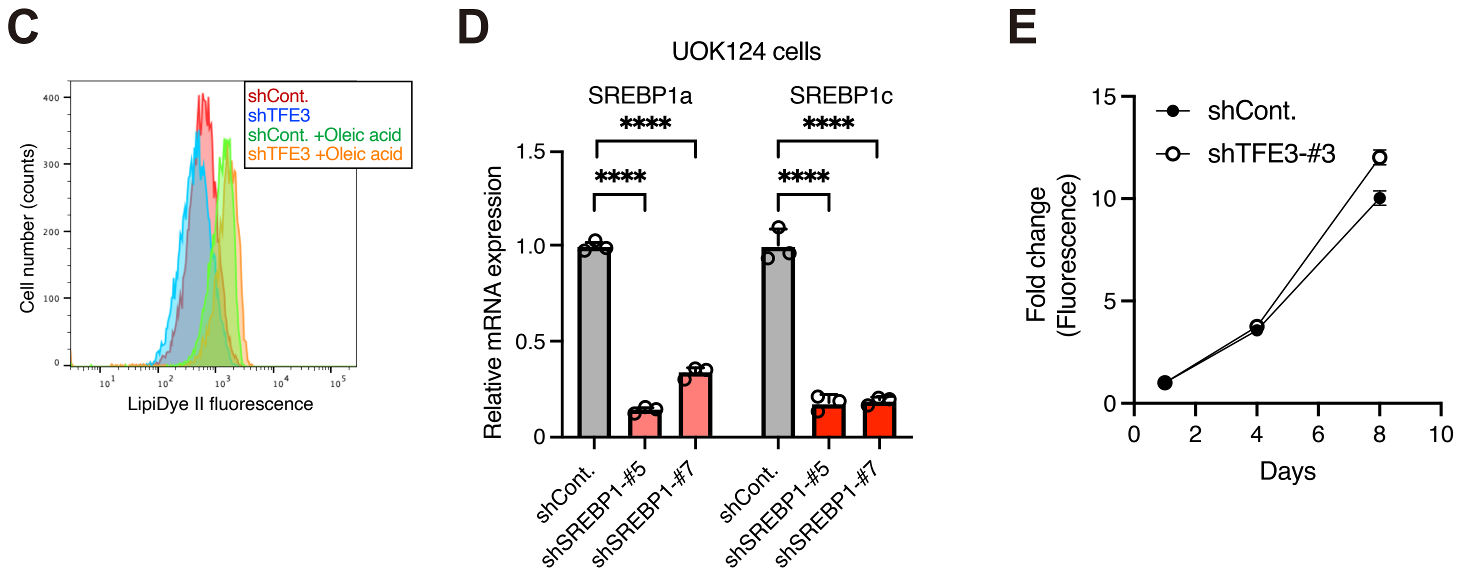


(A) Names and descriptions of the top-regulated genes involved in the ketone metabolic process in *PRCC-TFE3* KI kidneys, as shown in Figure 6A.

(B) RT-qPCR analysis of HIF1α in the PRCC-TFE3 inducible HK2 cells with HIF1α knockdown (n = 3). Data are means ± SD. ****$p < 0.0001$ (Welch's t-test)

(C) Representative FACS histogram showing LipidDyeII fluorescence, related to Figure 6D.

(D) RT-qPCR analysis of SREBP1a and SREBP1c in in the patient-derived PRCC-TFE3 RCC cell line (UOK124) with PRCC-TFE3 knockdown (n = 3).

Data are means ± SD. ****$p < 0.0001$ (The one-way ANOVA followed by the Dunnett post-hoc test)

(E) UOK124 cells expressing shCont or shTFE3 were cultured in 2D for the indicated times. Proliferation was normalized using resazurin fluorescence relative to day 1.

**Fig. S7**
