# Supplementary material for: HIF1α Plays a Crucial Role in the Development of TFE3–Rearranged Renal Cell Carcinoma by Orchestrating a Metabolic Shift Toward Fatty Acid Synthesis
Source: Genes Cells. 2025 Jan 14;30(1):e13195. doi: 10.1111/gtc.13195 (PMC11729263; doi:10.1111/gtc.13195)
Supplement: Supplementary file 8 — Figure S8. [file GTC-30-0-s005.pdf]

Srebp1 expression in PRCC-TFE3 mouse kidneys

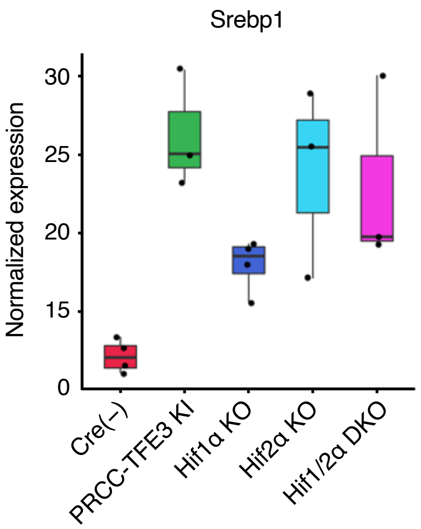

Normalized Srebp1 expression levels from mouse RNA-seq data presented in Figure 5.

Fig. S8
