# Supplementary material for: HIF1α Plays a Crucial Role in the Development of TFE3–Rearranged Renal Cell Carcinoma by Orchestrating a Metabolic Shift Toward Fatty Acid Synthesis
Source: Genes Cells. 2025 Jan 14;30(1):e13195. doi: 10.1111/gtc.13195 (PMC11729263; doi:10.1111/gtc.13195)
Supplement: Supplementary file 9 — Figure S9. [file GTC-30-0-s004.pdf]

TFE3 knockdown effect on HIF1α and HIF2α expression in UOK124 cells

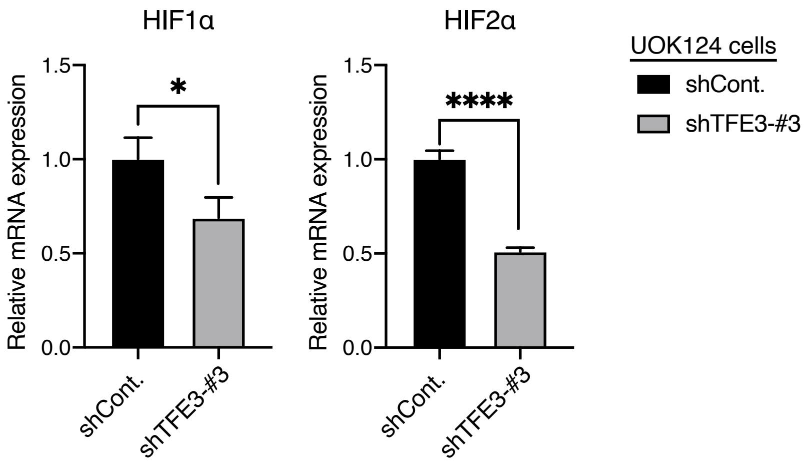

RT-qPCR analysis of HIF1α and HIF2α expression in patient-derived PRCC-TFE3 RCC cell lines (UOK124) with shControl and shTFE3 knockdown.

Fig. S9
